# Short Antimicrobial Peptide Derived from the Venom Gland Transcriptome of *Pamphobeteus verdolaga* Increases Gentamicin Susceptibility of Multidrug-Resistant *Klebsiella pneumoniae*

**DOI:** 10.3390/antibiotics13010006

**Published:** 2023-12-20

**Authors:** Cristian Salinas-Restrepo, Ana María Naranjo-Duran, Juan Quintana, Julio Bueno, Fanny Guzman, Lina M. Hoyos Palacio, Cesar Segura

**Affiliations:** 1 Grupo Toxinología, Alternativas Terapéuticas y Alimentarias, Facultad de Ciencias Farmacéuticas y Alimentarias, Universidad de Antioquia, Medellín 050012, Colombia; cristian.salinas@udea.edu.co (C.S.-R.); amaria.naranjo@udea.edu.co (A.M.N.-D.); 2 Facultad de Medicina, Universidad Cooperativa de Colombia, Medellín 050012, Colombia; juan.quintanac@ucc.edu.co; 3 Grupo Reproducción, Facultad de Medicina, Universidad de Antioquia, Medellín 050012, Colombia; julio.bueno@udea.edu.co; 4 Núcleo Biotecnología Curauma (NBC), Pontificia Universidad Católica de Valparaíso, Valparaíso 3100000, Chile; fanny.guzman@pucv.cl; 5 Escuela de Ciencias de la Salud, Grupo de Investigación Biología de Sistemas, Universidad Pontificia Bolivariana, Medellín 050031, Colombia; lina.hoyos@upb.edu.co; 6 Grupo Malaria, Facultad de Medicina, Universidad de Antioquia, Medellín 050012, Colombia

**Keywords:** *Pamphobeteus verdolaga*, antimicrobial peptide, *Escherichia coli*, *Staphylococcus aureus*, *Pseudomonas aeruginosa*, *Klebsiella pneumoniae*, multidrug resistant, gentamicin

## Abstract

Infectious diseases account for nine percent of annual human deaths, and the widespread emergence of antimicrobial resistances threatens to significantly increase this number in the coming decades. The prospect of antimicrobial peptides (AMPs) derived from venomous animals presents an interesting alternative for developing novel active pharmaceutical ingredients (APIs). Small, cationic and amphiphilic peptides were predicted from the venom gland transcriptome of *Pamphobeteus verdolaga* using a custom database of the arthropod’s AMPs. Ninety-four candidates were chemically synthesized and screened against ATCC^®^ strains of *Escherichia coli* and *Staphylococcus aureus*. Among them, one AMP, named PvAMP66, showed broad-spectrum antimicrobial properties with selectivity towards Gram-negative bacteria. It also exhibited activity against *Pseudomonas aeruginosa*, as well as both an ATCC^®^ and a clinically isolated multidrug-resistant (MDR) strain of *K. pneumoniae*. The scanning electron microscopy analysis revealed that PvAMP66 induced morphological changes of the MDR *K. pneumoniae* strain suggesting a potential “carpet model” mechanism of action. The isobologram analysis showed an additive interaction between PvAMP66 and gentamicin in inhibiting the growth of MDR *K. pneumoniae*, leading to a ten-fold reduction in gentamicin’s effective concentration. A cytotoxicity against erythrocytes or peripheral blood mononuclear cells was observed at concentrations three to thirteen-fold higher than those exhibited against the evaluated bacterial strains. This evidence suggests that PvAMP66 can serve as a template for the development of AMPs with enhanced activity and deserves further pre-clinical studies as an API in combination therapy.

## 1. Introduction

Infectious diseases significantly contribute to the global burden of morbidity and mortality. These are responsible for a noteworthy proportion of annual human fatalities, with approximately nine percent attributed to lower respiratory tract infections, intestinal infections, malaria, or tuberculosis [1]. Moreover, secondary infections in postoperative wounds or burns, infections in transplant or cancer patients, and foodborne illnesses significantly diminish the quality of life for affected individuals [2,3,4]. While several sanitary measures and the use of antibiotics have significantly reduced the impact of infectious diseases since the second half of the 20th century, the improper use and overuse of these medications have exacerbated the problem of antimicrobial resistance [5,6]. Challenges in describing new active molecules, development timelines exceeding ten years, low approval rates from regulatory authorities, and short product life cycles due to the likely emergence of resistance have significantly contributed to a dearth in the development of novel antimicrobials since the 1980s [3,7,8]. Today, it is estimated that over 700,000 human deaths are annually related to infections caused by antibiotic-resistant bacteria [9]. It is projected that by 2050 the annual number of human deaths caused by antimicrobial-resistant bacteria will exceed ten million [10]. Infectious diseases could potentially become the leading cause of human death by the middle of this century. Bacteria within the ESKAPE pathogen group (i.e., *Enterobacter* spp., *Staphylococcus aureus*, *Klebsiella pneumoniae*, *Acinetobacter baumannii*, *Pseudomonas aeruginosa*, and *Enterococcus* spp.) are a focal point of public health concerns because of their involvement in nosocomial infections and the alarming prevalence of their resistance to last-resort antibiotics [7,11,12,13,14]. Therefore, there is an urgent need for the development of novel antimicrobials targeting these bacterial species.

Antimicrobial peptides (AMPs) serve as the first line of defense in the innate immune response against bacterial infections [15]. AMPs play a crucial role in controlling bacterial loads, and can be produced in the absence of an infection through the proteolytic degradation (non-ribosomal production) of proteins unrelated to immunity, such as histones or casein [16,17]. Their primary mechanism of action involves disrupting membrane integrity, achieved either through pore formation (as observed in the barrel-stave or toroidal pore models) or through a detergent-like activity (carpet model). This disruption leads to cytoplasmic content leakage, cell membrane depolarization, and bacterial death [18]. Antibodies and bacteriophages are emerging as novel active pharmaceutical ingredients (APIs) in antimicrobial development [7]. On the other hand, AMPs, offer advantages, such as lower production costs and a reduced likelihood of resistance compared to antibiotics [19,20,21,22,23,24]. These factors make AMPs highly promising tools against antimicrobial resistances.

*Pamphobeteus verdolaga* (*Araneae: Theraphosidae*) is a spider endemic to Colombia [25]. A proteomic analysis of *P. verdolaga’s* venom has identified peptides with potential antimicrobial properties associated with their cationic and amphiphilic characteristics [26]. Therefore, the previously reported venom gland transcriptome of *P. verdolaga* [27] was examined to identify its peptides with hypothetical antimicrobial activity. The objectives of this study were: first, to bioinformatically identify and chemically synthesize the short, cationic and, amphiphilic peptides showing homology to the previously reported *Arthropoda* AMPs; second, to assess their antimicrobial activity against bacteria species from the ESKAPE group; third, to evaluate the activity of the most potent peptide against a clinically isolated multidrug-resistant strain of *K. pneumoniae* in combination with gentamicin; and fourth, to evaluate its cytotoxicity in vitro.

## 2. Results

### 2.1. Prediction and Synthesis of Short Cationic Hypothetical AMPs in P. verdolaga’s Transcriptome

The venom gland transcriptome of *P. verdolaga* [27] was examined to identify hypothetical AMPs. For this purpose, six peptide repositories and the PubMed search engine were searched for the cationic peptides described in venomous arthropods, which had lengths of fewer than 35 amino acids and were reported to exhibit experimental antimicrobial activity towards *E. coli* or *S. aureus*. The resulting custom database (cDB) comprised 197 non-redundant peptides from 68 arthropod species (Supplementary Table S1). This cDB was compared to the 18,338 genes from the venom gland transcriptome using the software BLAST+ v2.9.0 [28] and HMMER3 v3.2.1 [29]. The BLAST+ and HMMER3 strategies allowed the identification of 86 and 8 hypothetical AMPs, respectively (Supplementary Table S2). These peptides were associated with 90 *P. verdolaga* genes, had lengths ranging from 7 to 19 amino acids, carried a charge of +2 to +7 at pH 7.20, displayed amphipathic indexes between 0.41 and 2.70, and exhibited sequence identities to their homologous cDB sequences ranging from 50 to 100% (Supplementary Table S2). The candidate peptides were chemically synthesized through solid-phase peptide synthesis (SPPS) using an F-moc based methodology. Prior to solid phase extraction (SPE) purification, the identity and purity of the peptides were assessed. An LC/MS assessment enabled the identification of base peaks that corresponded to the expected *m*/*z* ratios (Supplementary Table S3). Furthermore, the base peak signals corresponded to the highest peak identified through RP-HPLC, indicating relative purities above 70%. For peptides capable of forming disulfide bridges, the presence of two peaks in the chromatograms indicated two oxidation stages (Supplementary Figure S1). Once their identity was confirmed, the peptides were subjected to oxidation and/or purification through SPE. Subsequently, their activity was screened against ATCC^®^ strains of *E. coli* and *S. aureus*.

### 2.2. Screening the Potential Antimicrobial Activity of P. verdolaga’s Hypothetical AMPs

Activity screening was conducted using the broth microdilution method at a fixed peptide concentration of 30 µM against *E. coli* ATCC^®^ 8739 and 25922, as well as *S. aureus* ATCC^®^ 6538 and 29213. The bacterial density used for screening was approximately 6 × 10^6^ CFU/mL [30]. Peptide activity measurements were conducted in terms of the growth inhibition percentage. No dose-response curves were generated during the screening phase as a resource optimization strategy in the initial activity evaluation. The hypothetical AMPs from *P. verdolaga* exhibited growth inhibitions to *E. coli* ATCC^®^ 8739 and *E. coli* ATCC^®^ 25922, ranging from 1% to 94% and 4% to 75%, respectively (Table 1). For *S. aureus* ATCC^®^ 6538 and *S. aureus* ATCC^®^ 29213, growth inhibition ranged from 0% to 140% and 0% to 98%, respectively (Table 1). Of the 94 peptides, 17 exhibited significant growth inhibition to at least one bacterial strain compared to the negative growth inhibition control of PBS (Table 1). Peptides PvAMP7, PvAMP32, PvAMP66, PvAMP82, PvAMP164, PvAMP172, PvAMP177, and PvAMP183 exhibited significant growth inhibition against both *E. coli* strains, classifying them as antimicrobials with selectivity towards Gram-negative bacteria (Table 1). Additionally, peptides PvAMP7, PvAMP32, PvAMP66, PvAMP69, PvAMP82, and PvAMP164 exhibited significant growth inhibition against both *S. aureus* strains, classifying them as antimicrobials with selectivity towards Gram-positive bacteria (Table 1). The AMPs exhibited amino acid homology to various toxins, including the M-zodatoxin-Lt1a toxin (Latarcin-1 or Ltc1) from the spider species *Lachesana tarabaevi*; the synthetic peptides Gomesin c(1-18)[Gln1] and cGomesin, derived from the spider species *Acanthoscurria gomesiana*; the synthetic peptides IsCT-P and IsCT [EK7, GP8, SK11] from the scorpion species *Opisthacanthus madagascariensis*; the Androcitin toxin from the scorpion species *Androctonus australis*; and the Scolopendin 2 toxin from the scolopendra species *Scolopendra subspinipes mutilans* (Supplementary Table S2).

### 2.3. MIC, MBC, and IC_50_ Values of P. verdolaga’s AMPs against ESKAPE Group Bacteria

The nine AMPs identified in the initial screening were evaluated at concentrations between 1.56–200 µM, against *E. coli* ATCC^®^ 25922, *S. aureus* ATCC^®^ 29213, *E. faecalis* ATCC^®^ 49532, *P. aeruginosa* ATCC^®^ 9027, and *K. pneumoniae* ATCC^®^ BAA-1705 (Supplementary Figures S2–S6). All peptides exhibited minimum inhibitory concentrations (MICs) against *E. coli*, with values ranging from 25–200 µM (Table 2). Only PvAMP32, PvAMP66, and PvAMP172 exhibited activity against *S. aureus*, with MICs of 25–100 µM (Table 2). None of the peptides exhibited activity against *E. faecalis*, but all peptides except PvAMP7 exhibited activity against *P. aeruginosa* at concentrations of 12.50–200 µM (Table 2). PvAMP32, PvAMP66, PvAMP82, PvAMP164, PvAMP172, PvAMP177, and PvAMP183 exhibited minimum bactericidal concentrations (MBCs) against both *E. coli* and *P. aeruginosa* at concentrations close to their MICs (Table 2). Only PvAMP66 and PvAMP172 reached the MBC for *S. aureus* at 50 µM and 100 µM, respectively (Table 2). This trend suggests that *P. verdolaga’s* peptides have greater selectivity for Gram-negative bacteria. Interestingly, PvAMP66 was the only peptide that exhibited activity against *K. pneumoniae*, with a MIC of 100 µM (Table 2). PvAMP66 exhibited broad-spectrum activity with an average MIC of 46.87 µM against both Gram-positive and Gram-negative bacteria representative of the ESKAPE group.

### 2.4. Assessing the Susceptibility of a Multidrug-Resistant K. pneumoniae Strain to PvAMP66, and Its Interaction with Gentamicin

**Figure 1:**
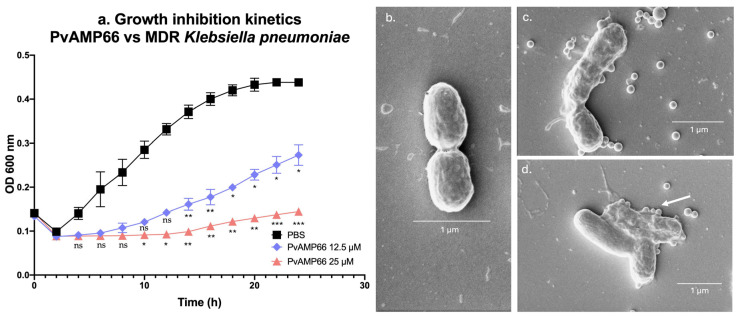
Activity of PvAMP66 against MDR *K. pneumoniae*. (**a**) Growth inhibition kinetics. Morphological changes: (**b**) PBS. (**c**) PvAMP66 at 25 µM. (**d**) PvAMP66 at 100 µM. Note: ns (not significant) = *p* > 0.05; * = *p* ≤ 0.05; ** = *p* ≤ 0.01; *** *p* ≤ 0.001.

A multidrug-resistant (MDR) strain of *K. pneumoniae* was identified and isolated from a tertiary-level hospital in Medellin, Colombia, using the VITEK-2 system (bioMérieux, Marcy-l’Étoile, France). The MDR strain was evaluated against several antibiotics at concentrations of 1.56–100 μM. The clinical isolate exhibited a complete resistance to streptomycin, penicillin, ampicillin, dicloxacillin, and oxytetracycline (Supplementary Figure S7b–f), with a maximum growth inhibition of 20% at 100 μM [30]. In contrast, the strain was susceptible to doxycycline (Supplementary Figure S7g) and chloramphenicol (Supplementary Figure S7h), with MICs of 50 μM and 100 µM, respectively (Table 3) [30]. Additionally, the strain exhibited a reduced susceptibility to gentamicin, with an MIC between 21.50 μM and 86 μM (Table 3) (Supplementary Figure S7a) [30]. All of *P. verdolaga*’s AMPs were evaluated against the clinical isolate at concentrations of 1.56–200 µM (Supplementary Figure S8). However, only PvAMP66 exhibited antimicrobial activity, with a MIC of 25 µM and an IC_50_ of 15.80 µM (Table 3). Interestingly, no bactericidal activity was observed at the evaluated concentrations. A growth inhibition kinetic assay was conducted. PvAMP66 exhibited a significant growth inhibition to MDR *K. pneumoniae* after 14 h of incubation at a concentration close to the IC_50_ value (Figure 1a). At the MIC, PvAMP66 induced minor changes in OD_600nm_ from 2 to 24 h post-incubation, but significant growth inhibition was evident after 10 h (Figure 1a). A scanning electron microscopy (SEM) revealed that PvAMP66 induced notable morphological changes in MDR *K. pneumoniae* at both 25 μM (Figure 1c) and 100 μM (Figure 1d), compared to the PBS negative control (Figure 1b). These changes included a “blebbing” effect, and at 100 μM, a total loss of membrane integrity was observed (Figure 1d). These morphological alterations strongly suggest that PvAMP66’s antimicrobial activity is likely mediated through interactions with the bacterial membrane. All of *P. verdolaga’s* peptides at 100 μM induced morphological changes in the MDR *K. pneumoniae*, indicating membrane interactions, even though no significant inhibition was observed (Supplementary Figure S9).

**Figure 2:**
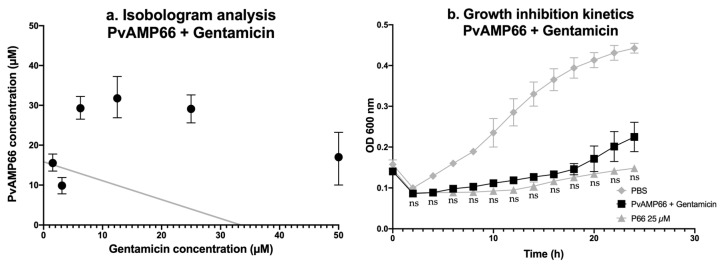
Interaction between PvAMP66 and Gentamicin: (**a**) Isobologram analysis. (**b**) Kinetic of the growth inhibition for the concentration that achieved MIC. Note: ns (not significant) = *p* > 0.05.

Given the intermediate resistance to gentamicin, an isobologram analysis was used as a proof-of-concept to evaluate the interaction between PvAMP66 and the antibiotic [31]. The growth inhibition of the clinical isolate was measured using peptide concentrations of 0.10–100 µM, and gentamicin concentrations of 0.10–50 µM (Supplementary Figure S10). Combined IC_50_ values were calculated and plotted in a graph with a secant line representing the IC_50_ values for PvAMP66 and gentamicin when used alone (Figure 2a). The IC_50_ values showed a bell-shape distribution, with most values above the secant line, indicating an antagonistic interaction between the two molecules. However, two datasets were located adjacent to the secant line, suggesting an additive interaction between the compounds (Figure 2a). One of these data points represented a 10.68-fold reduction in the IC_50_ for gentamicin (3.12 µM) and a 60% reduction in the IC_50_ for PvAMP66 (9.85 µM) (Supplementary Table S4). The concentrations adjacent to the aforementioned data point were evaluated. The combination of 4.70 µM gentamicin and 12.50 µM PvAMP66 achieved the MIC against *K. pneumoniae* MDR (Figure 2b). This represented a 10.64-fold reduction in the MIC for gentamicin and a 2-fold reduction for PvAMP66. Additionally, a Fractional Inhibitory Concentration Index (FICI) of 0.59 was obtained [32]. Furthermore, the kinetics of growth inhibition at this concentration showed no significant difference compared to the use of PvAMP66 alone (Figure 2b). This proof-of-concept assay suggests that the combined use of PvAMP66 and gentamicin can increase the clinical isolate’s susceptibility to gentamicin (MIC 4.70 < 21.5 µM [30]).

### 2.5. Assessing the Cytotoxicity of PvAMP66

**Figure 3:**
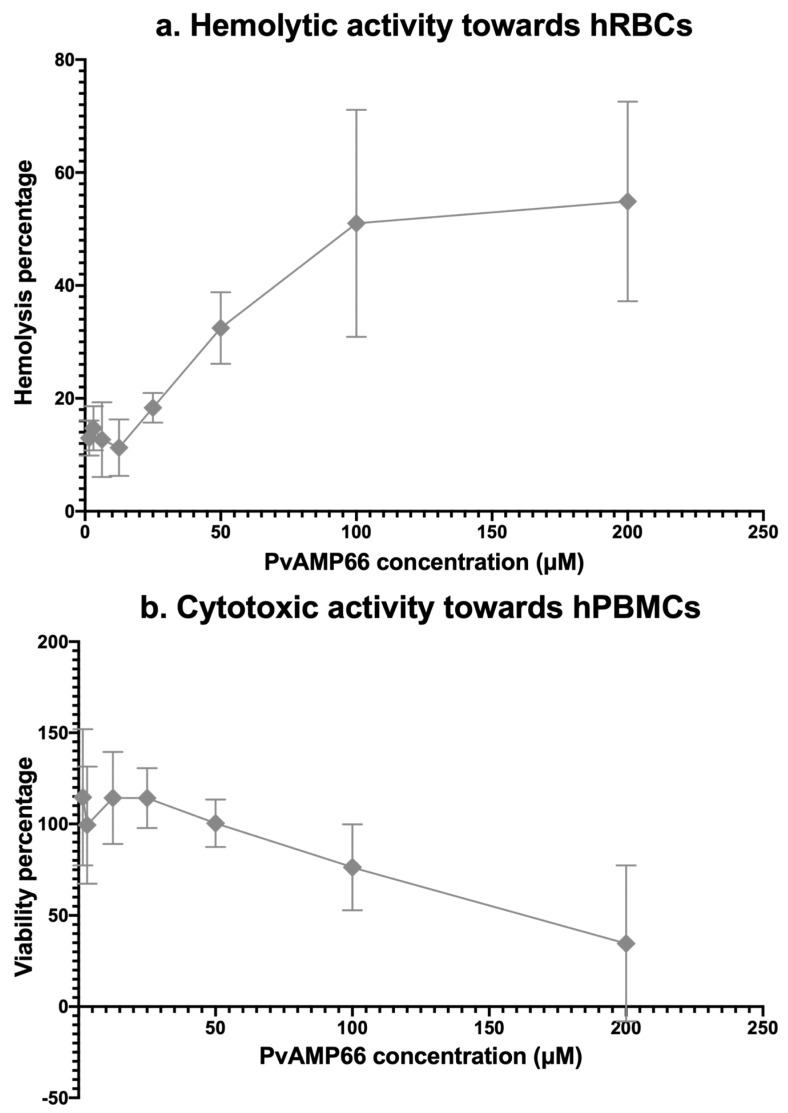
Dose-response curves of PvAMP66 against: (**a**) Human red blood cells. (**b**) Human peripheral blood mononuclear cells.

The cytotoxicity of PvAMP66 was assessed in vitro against human red blood cells (hRBCs) and human peripheral blood mononuclear cells (hPBMCs) using concentrations of 1.56–200 µM. At the highest concentration, PvAMP66 induced 60% hemolysis in human erythrocytes (Figure 3a), and a decrease in viability of approximately 40% in the hPBMCs (Figure 3b). A logistic regression of the data revealed that the Hemolytic Concentration 50 (HC_50_) and Cytotoxic Concentration 50 (CC_50_) were 132.96 µM and 172.03 µM, respectively (Table 4). Ratios between CC_50_ or HC_50_, and the IC_50_ values registered for the bacterial strains, indicate that PvAMP66 has higher selectivity for interacting with prokaryotic membranes (Table 4). PvAMP66 exhibits the highest selectivity towards *P. aeruginosa* (Indexes between 10 and 13), followed by the MDR *K. pneumoniae* strain (Indexes between 8 and 10), and then *S. aureus* (Indexes between 4 and 6). Selectivity indexes ranging from three to thirteen may suggest that PvAMP66 could function as a compound with a narrow therapeutic index [33,34,35].

## 3. Discussion

The genus *Pamphobeteus* (*Araneae: Theraphosidae*) comprises 33 tarantula species endemic to South America. *P. verdolaga* is the first species described in Colombia since *Pamphobeteus insignis* and *Pamphobeteus ornatus* by Pocock in 1903 [25,36]. Theraphosids rely uniquely on their venom for predation, thus, tarantula venom contains peptides and proteins of interest to biotechnology [27,37,38]. Antimicrobial peptides in spider venom are thought to facilitate the diffusion of neurotoxic peptides and other high molecular mass toxins, and enforce the sterility of the chelicerae [39,40]. Due to technical challenges in venom characterization [41], lack of nucleotide data [27], and the absence of bioinformatic tools for tissues such as venom glands, there is a scarcity of reported data on AMPs from arachnids in general [42,43]. Therefore, owing to their complexity, spider venoms are enticing and unexplored alternatives for prospecting novel AMPs.

### 3.1. Prospecting Hypothetical AMPs from the Venom Gland Transcriptome of P. verdolaga

The search for novel AMPs from the venom gland transcriptome of *P. verdolaga* focused on identifying small, cationic, and amphiphilic peptides. A shared identity in the primary structure of proteins or peptides is a strong indicator of a common origin and/or similar function [44]. Due to the scarcity of reported AMPs from spiders, hypothetical AMPs from the venom gland of *P. verdolaga* were predicted based on homology with AMPs described in venomous species from the classes *Arachnida* and *Insecta*, the *Scorpiones* order, as well as the subphylum *Myriapoda*. A custom database (cDB) of peptides was created using public databases and PubMed. This database was filtered based on redundancy, charge, length, and in vitro activity. The extracted peptides exhibited activity against either *E. coli* or *S. aureus* between 0.05 µM and 500 µM. To increase the likelihood of obtaining at least one active candidate AMP, the most active AMPs from the cDB were used as templates. A cutoff of 15 µM was employed as this represented the 50th percentile of activity (Supplementary Table S1).

Candidate peptides were predicted using BLAST+ and HMMER3 [44]. Unlike pairwise alignment (BLAST+) methodologies, which use BLOSUM or PAM matrixes to account for the probability of amino acid substitution, a HMM profile prediction (HMMER3) is more restrictive, considering only the states of “match”, “insertion”, and “gap” [45,46]. To ensure a minimum of 50% production yield by SPPS, a cutoff of 35 amino acids was used for the maximum peptide length [47]. A minimum length of seven amino acids was used to ensure the probable formation of either barrel-stave or toroidal pores on a bacterial membrane [48,49]. This approach led to the identification of 94 candidate peptides (Supplementary Table S2), with only eight identified through HMMER3. Most AMP prospecting studies from venomous organisms employ bottom-up proteomic strategies [50,51]. Nevertheless, prospecting methodologies similar to the one employed here have also been utilized for the description of the AMPs Scolopendin 1 and 2 from *S. subspinipes mutilans* [52,53,54], over 112 hypothetical AMPs from the venom gland transcriptome of the ant *Odontomachus chelifer* [55], and the peptides Ak-N′ and Ak-N′m from the spider *Agelena koreana* [56]. To the best of our knowledge, this work represents the first report of its kind on spiders of the *Pamphobeteus* genus. However, it is important to note that a major limitation of this study is the absence of confirmation of either the associated transcripts or the candidate AMPs within the venom gland of *P. verdolaga*. Consequently, confirmation through methods like RT-PCR or LC/MS is mandatory in future studies.

### 3.2. Activity of the Hypothetical AMPs from P. verdolaga

All candidate peptides were synthesized by SPPS in their amide form [57] and screened against the reference strains *E. coli* ATCC^®^ 8739, *S. aureus* ATCC^®^ 6538 [58], *E. coli* ATCC^®^ 25922, and *S. aureus* ATCC^®^ 29213 [30]. Antimicrobial activity can vary by up to 16-fold among different strains of the same bacterial species [59,60]. Therefore, only peptides that exhibited activity against both reference strains of *E. coli* or *S. aureus* were considered antimicrobial. Peptides were initially screened at 15 μM, the cDB cutoff concentration, with only PvAMP66 exhibiting significant growth inhibition. The concentration was doubled to 30 μM to broaden AMP detection. Nine peptides, accounting for 9.60% of the evaluated molecules, exhibited antimicrobial activity. Despite the use of AMPs from various arthropod species to create the cDB (Supplementary Table S1), the peptides identified as antimicrobials exhibited homology with AMPs described in the *Scorpiones* and *Araneae* orders, and *Scolopendra* genera (Supplementary Table S2). This is likely due to the hypothesized closer phylogenetic relationship between the *Chelicerata* and *Myriapoda* subphyla relative to *Hexapoda* [61].

ESKAPE pathogens pose a serious public health threat due to their high virulence and their role in nosocomial infections [7,62]. Infections caused by ESKAPE pathogens lead to longer hospital stays, higher healthcare costs, and higher mortality rates than infections caused by non-ESKAPE pathogens [63]. Developing antimicrobials effective against Gram-negative bacteria is generally considered to be more challenging because of their reduced outer membrane permeability [7]. Nevertheless, all of *P. verdolaga’s* peptides, except PvAMP7 and PvAMP69, exhibited greater activity against *E. coli* and *P. aeruginosa* (Table 2). This trend is likely due to the similarities in the percentages of phosphatidylglycerol (PG) and cardiolipin (CL) in the membranes of both species [64,65,66]. In contrast, only PvAMP66 exhibited activity against *K. pneumoniae* ATCC^®^ BAA-1705, which harbors the *bla_KPC-2_* gene and exhibits a mild biofilm formation [67]. The observed low AMP susceptibility might be related to the production of capsular polysaccharide [68]. Among the Gram-positive bacteria tested, only PvAMP32, PvAMP66, and PvAMP172 exhibited activity against *S. aureus*, while none of the peptides showed activity against *E. faecalis*. *E. faecalis* possesses constitutive resistance to membrane damage by incorporating exogenous fatty acids and anionic phospholipids into its cell membrane when exposed to immune system AMPs (e.g., LL-37) or antibiotic lipopeptides (e.g., daptomycin or vancomycin). This response is initiated by the LiaX protein, which activates the LiaSFR system [69,70,71]. A similar mechanism, the VraTSR pathway, has been reported in *S. aureus* [72]. These unique mechanisms could explain the limited activity of *P. verdolaga’s* AMPs against *E. faecalis* and *S. aureus*, respectively.

### 3.3. PvAMP66 Enhances Gentamicin Inhibition of MDR K. pneumoniae Growth

*K. pneumoniae* is a major contributor to nosocomial infections, with particular concern surrounding the KPC and ESBL phenotypes [12,73]. Multidrug-resistant strains of *K. pneumoniae* have demonstrated a cross-resistance between AMPs and conventional antibiotics. This cross-resistance is facilitated by the AcrAB efflux pump, which mediates resistance to AMPs like HBD-1, HBD-2, and HNP-1, as well as quinolones, erythromycin, tetracycline, chloramphenicol, or aminoglycosides [74]. Furthermore, reduced LL-37 activity has been associated with a resistance to colistin or polymyxin B, a phenomenon mediated by the *mgrB*–PhoP/Q axis, which diminishes AMP permeability [75,76]. Only PvAMP66 exhibited antimicrobial activity against the multidrug-resistant clinical isolate of *K. pneumoniae* (Supplementary Figure S8), with a bacteriostatic effect at all tested concentrations, as confirmed by the inhibition kinetics. The observed ‘blebbing effect’ (Figure 1c,d), and a length of approximately 25 to 28 Å [77], strongly suggest that PvAMP66’s antimicrobial effect is mediated through a carpet model mechanism of action [48,49]. Similar changes in morphology have been reported for IsCT (PvAMP66’s homolog) and peptide AMP-016 on *E. coli* [78,79]. However, given the intricate interactions occurring within the peptide/phospholipid/transmembrane-protein complexes, additional research is needed to fully elucidate the complete mechanism of action of PvAMP66.

Given the paucity of newly approved antimicrobial agents, it is noteworthy that 20% of the options in the clinical antimicrobial development pipeline are API combinations [7]. Additionally, the increasing prevalence of KPC and/or ESBL phenotypes has led to a greater reliance on aminoglycosides for the treatment of infections caused by MDR *K. pneumoniae* [80,81,82]. The evaluation of the interaction between the aminoglycoside gentamicin and PvAMP66 was prompted by the observed diminished susceptibility of MDR *K. pneumoniae* to this antibiotic (Table 3). The isobologram analysis suggested that the interaction between PvAMP66 and gentamicin was predominantly antagonistic (Figure 2a) (Supplementary Table S4) [30]. Gentamicin, possessing a charge of +5 at pH 7.2, gains access to the cytosol through electrostatic interactions with the teichoic acid, Lipopolysaccharide, PG, or CL present on the bacterial surface [83,84]. Therefore, the antagonistic interaction observed was likely mediated by the competition between these two molecules for the limited partial anionic charges available on the bacterial membrane of MDR *K. pneumoniae*. The isobologram analysis allowed the identification of a specific concentration range in which an additive interaction was observed for both compounds (Figure 2a). This effect was probably mediated by an enhanced entry of the antibiotic into the bacterial cytoplasm facilitated by the evident loss of membrane integrity induced by PvAMP66 (Figure 1c). FICI values below 0.50 are associated with synergistic interactions, while values between 0.50 and 1 are associated with additive interactions [32]. At the combined minimum inhibitory concentration, a FICI value of 0.59 was obtained, confirming the additive interaction of both molecules. Considering these observed interactions, PvAMP66 holds potential for further development as a type I adjuvant [30,85,86].

### 3.4. PvAMP66 Is P. verdolaga’s Most Active AMP

Amongst *P. verdolaga’s* AMPs, PvAMP66 exhibited the highest ratio between Trp and Lys, and the highest ratio between charge and amphiphilicity (Supplementary Table S2). These physicochemical traits are related to the enhanced antimicrobial activity due to the higher selectivity towards the bacterial membrane [78,87,88]. HC_50_ and CC_50_ values of 132.96 µM and 172.03 µM, respectively, were obtained for PvAMP66 (Table 4). PvAMP66 exhibited a higher selectivity for erythrocytes, which may be due to the higher percentage of phosphatidylserine on the inner membrane leaflet of human red blood cells (hRBCs) compared to lymphocytes, which make up about 70 to 90% of human peripheral blood mononuclear cells (hPBMCs) [89,90,91]. The therapeutic potential of an API is heavily contingent on the balance between activity and toxicity. The therapeutic index relies on distinct pharmacodynamic and pharmacokinetic profiles [33,35]. The determination of an in vitro selectivity index is a valuable tool to initially assess the therapeutic potential. PvAMP66 displayed a higher selectivity index for bacteria, ranging from three- to thirteen-fold times that of mammalian cells (Table 4). In contrast, LL-37, one of the most promising AMPs in the current pre-clinical and clinical development pipelines [7,92], can exhibit in vitro selectivity indexes that approximate one, depending on the chosen model [93,94,95]. Considering the observed activity profiles, PvAMP66 may potentially exhibit characteristics of a Narrow Therapeutic Index (NTID) compound when dealing with *E. coli* or *S. aureus*. However, it displays a higher safety profile when applied against *K. pneumoniae* or *P. aeruginosa* (Table 4).

High-throughput screening initiatives conducted by pharma companies have set MIC values of 8 µg/mL as approximate cut-off values for considering an antimicrobial as a promising candidate for the lead optimization phase [96,97]. Given its MIC values of 7.03 μg/mL against *E. coli* ATCC^®^ 25922, 3.51 μg/mL against *S. aureus* ATCC^®^ 29213, 1.77 μg/mL against *P. aeruginosa* ATCC^®^ 9027, and notably, 3.51 μg/mL against MDR *K. pneumoniae*, PvAMP66 can be considered as a prime template for the development of AMPs with enhanced activity (lead optimization). Furthermore, it can be considered for further pre-clinical development as an API in combinational therapy.

## 4. Materials and Methods

### 4.1. Reagents & Bacterial Strains

Fmoc-L-amino acids, Rink MBHA resin, bromophenol blue, N-[(1H-benzotriazol-1-yl)-(dimethylamino)methylene]-N-methylmethanaminium hexafluorophosphate N-oxide (HBTU), O-(Benzotriazol-1-yl)-N,N,N′,N′-tetramethyluroniumtetrafluoroborate (TBTU), N-[6-chloro(1H-benzotriazol-1-yl)-(dimethylamino)methylene]-N-methylmethanaminium hexafluorophosphate N-oxide (HCTU), N,N-diisopropylethylamine (DIPEA), OxymaPure, piperazine, N,N-dimethylformamide (DMF), isopropyl alcohol (IPA), anhydrous ethanol, dichloromethane (DCM), trifluoroacetic acid (TFA), triisopropylsilane (TIS), 2-mercaptoethanol, diethyl ether, solid phase extraction LiChrolut^®^ RP-18 cartridges, acetonitrile, guanidine-HCl, dimethyl sulfoxide (DMSO), ammonium hydroxide, sodium hydroxide, hydrochloric acid, sodium chloride, potassium chloride, sodium phosphate dibasic, potassium phosphate monobasic, chloramphenicol, penicillin, gentamicin sulfate, hydrogen peroxide 30%, Ficoll^®^, and grade I 70% glutaraldehyde were all obtained from Millipore-Sigma (Burlington, MA, USA). Sterile PBS pH 7.20 (Gibco), RPMI Media (Gibco), Trypan Blue (Gibco), Triton X-100, and 3-(4,5-dimethylthiazol-2-yl)-2,5-diphenyltetrazolium bromide (MTT) were obtained from ThermoFisher Scientific (Waltham, MA, USA). Mueller-Hinton Agar and Mueller-Hinton Broth were obtained from Scharlab (Barcelona, Catalonia, Spain). Plastic Petri dishes and 96-well culture plates were obtained from VWR (Radnor, PA, USA). The 50 mL conical polypropylene tubes and 0.50 mL polypropylene vials used were obtained from Eppendorf (Eppendorf Group, Hamburg, Germany). Ampicillin sodium salt was acquired from Research Organics (Cleveland, OH, USA). Streptomycin sulfate was acquired from MP Biomedicals (Solon, OH, USA). Bacterial cell lines derived from *E. coli* ATCC^®^ 8739 and ATCC^®^ 25922, *S. aureus* ATCC^®^ 6538 and ATCC^®^ 29213, *E. faecalis* ATCC^®^ 49532, *P. aeruginosa* ATCC^®^ 9027 as well as *K. pneumoniae* ATCC^®^ BAA-1705, were all obtained from Microbiologics (St. Cloud, MN, USA).

### 4.2. Creation of the Custom AMP Database and Prediction of Antimicrobial Peptides from the Venom Gland Transcriptome of P. verdolaga

The following databases were used: the Antimicrobial Peptide Database [98] (Available at https://aps.unmc.edu, accessed on 30 June 2019), the Collection of Antimicrobial Peptides [99] (Available at http://www.camp.bicnirrh.res.in, accessed on 30 June 2019), the Database of Antimicrobial Activity [100] (Available at https://dbaasp.org/home, accessed on 30 June 2019), Liking Antimicrobial Peptides [101] (Available at http://biotechlab.fudan.edu.cn/database/lamp/index.php, accessed on 30 June 2019), Yet Another Database of Antimicrobial Peptides [102] (Available at https://webs.iiitd.edu.in/raghava/satpdb/catalogs/yadamp/, accessed on 30 June 2019), the Arachnoserver [43] (Available at http://www.arachnoserver.org, accessed on 30 June 2019), and the PubMed search engine (Avaiable at http://pubmed.ncbi.nlm.nih.gov, accessed on 30 June 2019). The search was conducted for antimicrobial peptides related to the terms: Spider, Scorpion, Wasp, Ant, Bee, Scolopendra, Myriapod, Tick, and Arachnid, in combination with Venom and the terms: Antimicrobial, Cytolytic, Bacteriostatic, or Bactericidal. The resulting peptides were curated for duplicates and filtered so the peptides to be included in the custom database (cDB) were 7 to 35 amino acids in length, cationic at pH 7.20, and possessed experimental evidence of antimicrobial activity towards either *S. aureus* or *E. coli*; for peptides with multiple activity reports, MIC values were averaged. The peptides of the cDB were contrasted to the venom gland transcriptome of the *P. verdolaga* [27] (Available at DDBJ/EMBL/GenBank under the accession GIUY00000000) using the software BLAST+ v.2.9.0 [28] (Available at https://ftp.ncbi.nlm.nih.gov/blast/executables/blast+/LATEST/, accessed on 31 August 2019) and the software HMMER3 v3.2.1 [29] (Available at http://hmmer.org, accessed on 31 August 2019), after .msa files were created using the software Clustal Omega v1.2.4 (Available at https://github.com/GSLBiotech/clustal-omega, accessed on 31 August 2019). Redundant sequences were filtered using the software CD-HIT v4.8.1 [103] (Available at https://github.com/weizhongli/cdhit, accessed on 31 August 2019).

### 4.3. Synthesis, Purification, Disulfide Bridge Formation, and Quality Control of Candidate AMPs

Peptides were manually synthesized by solid-phase peptide synthesis (SPPS) following a standard Fmoc/tBu protocol [104] using Rink MBHA resin as solid support. In detail, all peptides were synthesized in parallel using 50 mg of resin (0.52 mmol-eq/g) (MC5 Micro Balance, Sartorius AG, Göttingen, Germany) in polypropylene tea bags. Deprotection was carried out under agitation at 300 rpm (AT) (SH-200, Cole-Parmer Instrument Company, Vernon Hills, IL, USA) and at room temperature (RT) in two 20-min cycles of 10% *w*/*v* piperazine in 10% *v*/*v* ethanol in DMF. Deprotection completion was monitored using a 1% *w*/*v* bromophenol blue solution in DMF. Both the Fmoc-L-amino acids and activators (HBTU and OxymaPure^®^) were used in a 10-fold excess for a final concentration of 0.26 M, while the DIPEA was used in a concentration of 0.52 M (MC5 Micro Balance, Sartorius AG, Göttingen, Germany). Five-hour coupling cycles were employed under AT and RT for HBTU. Coupling completion was monitored by the absence of bromophenol blue coloration. If color remained, a second and third coupling cycle was carried out for two and one hour, respectively (under AT and at RT), using either TBTU/OxymaPure^®^ or HCTU/OxymaPure^®^ at a concentration of 0.14 M in combination with DIPEA at a concentration of 0.26 M (MC5 Micro Balance, Sartorius AG, Göttingen, Germany). After the required elongation was achieved, peptides were cleaved from the solid support for two hours under AT and at RT using a 92.5:2.5:2.5:2.5 solution of TFA/TIS/2-mercaptoethanol/H_2_O, respectively. Peptides were removed from the cleavage solution by five cycles of cold (−20 °C) diethyl ether precipitation and centrifugation (2000× *g* for 15 min at 4 °C) (Universal 32 R, Hettich Group, Kirchlengern, Westphalia, Germany); the remaining diethyl ether was dried in a gas cabin (CEX 120, C4 Control de Contaminación SAS, Cali, Valle del Cauca, Colombia). Peptides were then dissolved in ultra-pure water, frozen at -70 °C (ULF 440 E Pro 2, EVERmed s.r.l., Motteggiana, Mantua, Italy) and lyophilized (FreeZone 12 L, Labconco Corporation, Kansas City, MO, USA) for storage.

The purity and identity verification of the peptides was carried out through RP-HPLC and LC/MS. Briefly, a 1 µg/µL stock of the peptides was prepared in a 0.05% *v*/*v* TFA water solution, and analyzed on a JASCO Corp HPLC (JASCO Corp, Tokyo, Japan) with an XBridge^TM^ BEH C18 column (Waters Corporation, Milford, Massachusets, USA). An amount of 100 µg of peptides were analyzed on a 0 to 70% 0.05% *v*/*v* TFA acetonitrile gradient at a 1 mL/min rate for nine minutes; peptide monitoring was conducted at 214 nm. Identity verification was carried on a LCMS-2020 ESI-MS (Shimadzu Corp., Kyoto, Japan) by the injection of 100 µg of peptide on positive ion mode at 4.5 kV and 350 °C; the run consisted of a 0 to 70% 0.05% *v*/*v* TFA acetonitrile gradient at a 1 mL/min for 20 min. The RP-HPLC data was acquired using JASCO Corp’s own proprietary software (ChromPass v1.7.403.1) while the LC/MS data acquisition and deconvolution were carried out using the LabSolutions SP3 software v5.43 (Shimadzu Corp., Kyoto, Japan).

After synthesis confirmation, peptides that allowed the formation of disulfide bridges were oxidized using a modified version of the protocol published by Zhang et al. [105]. Briefly, peptides were dissolved in a 5 mM Guanidine-HCl, 10% *v*/*v*, 10% *v*/*v* DMSO aqueous solution to a concentration of 0.20 mg/mL (MC5 Micro Balance, Sartorius AG, Göttingen, Germany); pH was adjusted to 7.00 with Ammonium hydroxide (Laqua PH1100, Horiba Scientific, Piscataway, NJ, USA). The oxidation was carried out at 300 rpm (SH-200, Cole-Parmer Instrument Company, Vernon Hills, IL, USA) for 16 h at room temperature. Following this, the peptides were frozen at −70 °C (ULF 440 E Pro 2, EVERmed s.r.l., Motteggiana, Mantua, Italy) and lyophilized (FreeZone 12 L, Labconco Corporation, Kansas City, MO, USA). Guanidine and DMSO were removed by solid phase extraction using LiChrolut^®^ RP-18 cartridges in acetonitrile *v*/*v* percentages between 0 and 100%, with 10% increments. The peptides that were not oxidized underwent the same procedure for further purification. There was no evaluation of disulfide bridge formation patterns or changes in mass due to possible oligomerization. Acetonitrile was removed under a gentle vacuum at 45 °C (Vacufuge plus, Eppendorf Group, Hamburg, Germany), and peptides were again frozen at −70 °C (ULF 440 E Pro 2, EVERmed s.r.l., Motteggiana, Mantua, Italy) and lyophilized (FreeZone 12 L, Labconco Corporation, Kansas City, MO, USA).

### 4.4. Evaluation of the Antimicrobial Activity of P. verdolaga’s AMPs

The evaluation of the antimicrobial activity of the peptides was conducted using the CLSI’s broth microdilution method for bacterial susceptibility testing [30]. For the screening phase, peptides were evaluated at a single 30 μM concentration, in triplicate and in three independent assays, against the *E. coli* ATCC^®^ 8739, *E. coli* ATCC^®^ 25922, *S. aureus* ATCC^®^ 6538, or *S. aureus* ATCC^®^ 29213 reference USP and CLSI strains [30,58]. For the determination of MIC, MBC, or IC_50_, peptides or antibiotics were evaluated in concentrations between 1.56–200 µM. These evaluations were conducted in triplicate and in three independent assays, against the *E. coli* ATCC^®^ 25922, *S. aureus* ATCC^®^ 29213, *E. faecalis* ATCC^®^ 49532, *P. aeruginosa* ATCC^®^ 9027, and *K. pneumoniae* ATCC^®^ BAA-1705, or the clinically isolated MDR strain. In detail, 1 mL of a glycerol stock solution of the ATCC^®^ or clinically isolated bacterial strain was added to 9 mL of Mueller-Hinton Broth and incubated for 18 h, 180 rpm and 37 °C (Incubator 1000, Heidolph Instruments, Schwabach, Germany). After the incubation, 1 mL of the previous solution was transferred to a new recipient containing 9 mL of fresh Mueller-Hinton broth and incubated for 4 h, 180 rpm and 37 °C (Incubator 1000, Heidolph Instruments, Schwabach, Germany). During the second incubation, the peptides or the antibiotics were dissolved in PBS pH 7.20 in concentrations between 19.53–2500 µM (MC5 Micro Balance, Sartorius AG, Göttingen, Germany). An amount of 10 µL of either peptide or antibiotic was dispensed in triplicate in 96-well flat bottom culture plates, along with 10 µL of PBS (Negative activity control), BTM-P1 (Positive activity control), or 10 µL of Mueller-Hinton broth (Blank); then, 110 µL of Mueller-Hinton broth was poured in all wells. Following this, the bacteria were suspended in Mueller-Hinton broth up to a concentration of approximately 1.50 × 10^8^ CFU/mL, as measured by optical density at 600 nm between 0.06 to 0.11 (0.5 McFarland standard), in a Multiskan SkyHigh spectrophotometer (ThermoFisher Scientific, Waltham, MA, USA). Amounts of 5 µL of the 1.50 × 10^8^ CFU/mL suspension were added to each well (except for the blank) for a final well concentration of approximately 6 × 10^6^ CFU/mL. The plate was incubated for 24 h at 37 °C (BE 500, Memmert GmbH, Büchenbach, Germany) and optical density was measured at 600 nm in a Multiskan SkyHigh spectrophotometer (ThermoFisher Scientific, Waltham, MI, USA). Following the measurement of optical density, plates were visually inspected to determine the MIC. Subsequently, 100 µL of the concentration associated to the MIC and 100 µL of the next higher concentration were added to Petri dishes containing Mueller-Hinton Agar. The dishes were then incubated for 24 h at 37 °C (BE 500, Memmert GmbH, Büchenbach, Germany). After incubation, the Petri dishes were visually inspected, and the MBC was determined in the absence of growth. IC_50_ values were interpolated from regressions with correlation values above 0.70. Growth inhibition percentages were calculated as follows:

$$Growth\;inhibition\;\%=\left(1-\frac{{Abs}_{PEPTIDE\;or\;ANTIBIOTIC}-{Median\;Abs}_{BLANK}}{{Median\;Abs}_{PBS}-{Median\;Abs}_{BLANK}}\right)\times 100$$

For the isobologram analysis concentrations, ranges of 1.56 µM to 200 µM of the peptide and 0.07 µM to 100 µM of the gentamicin were used. The experiments were conducted in triplicate and repeated in two independent assays. The IC_50_ for each combination was determined using either logarithmic or linear regression. Then, when achieving 50% growth inhibition, the concentrations of the peptide and gentamicin were recorded and used to create an isobologram graph with a ‘peptide concentration’ vs. ‘gentamicin concentration’ plot. Within the graph, both axes were connected in line with an origin at the IC_50_ value of the peptide and gentamicin alone in the Y and X-axis, respectively. The presence of data distributed below the line indicated a synergistic interaction, data distributed above or close to the line in a parallel fashion represented an additive interaction, and finally, data distributed above the line represented an antagonistic interaction [31]. The fractional inhibitory concentration index [32] was calculated as follows:

$$FICI=\frac{{Combined\;MIC}_{Antibiotic}}{{MIC}_{Antibiotic}}+\frac{{Combined\;MIC}_{Peptide}}{{MIC}_{Peptide}}$$

### 4.5. Scanning Electron Microscopy Analysis

The effect of the *P. verdolaga’s* AMPs on the bacterial surface of the MDR *K. pneumoniae* strain was evaluated using Scanning Electron Microscopy with an Apreo 2 S LoVac microscope (Thermo Fisher Scientific, Waltham, MA, USA). The MDR *K. pneumoniae* cells were incubated with all peptides at a single concentration of 100 µM, as previously mentioned. After incubation, the contents from the triplicate wells for each peptide were pooled in a 0.50 mL polypropylene tube, and 100 µL were placed onto sterile glass slides to air dry at room temperature. Subsequently, slides were submerged individually in 50 mL of a fixating solution (2.5% *v*/*v* glutaraldehyde solution in PBS pH 7.20) in a desiccator for 1 h. After fixation, the bacteria were dehydrated in a series of increasing ethanol concentrations (10, 25, 50, 75, and 100% *v*/*v*) in PBS pH 7.20 inside a desiccator. Each concentration percentage was allowed to contact the slides for 15 min at RT. After the highest ethanol percentage was reached, the samples were stored in closed Petri dishes until they were ready for examination. For image capture, the samples were gold-coated using a DC Magnetron Cathode apparatus (Denton Vacuum Desk IV). Then, samples were examined using the electron microscope under a vacuum of 1 bar, with an accelerating voltage of 1.00 kV, a current between 25–50 pA, and a working distance of 5.60 mm. All images were acquired using Xt Microscope Version 23.2.0 software (Thermo Fisher Scientific, Waltham, MA, USA).

### 4.6. Evaluation of the Hemolytic Activity and Cytotoxicity of P. verdolaga’s AMPs

Human red blood cells (hRBCs) were obtained from the Blood Bank of the School of Microbiology of the University of Antioquia in Medellín, Colombia. Ten mL of hRBCs were diluted in 10 mL of PSB pH 7.20, mixed by inversion, and centrifuged five times at 4 °C, 1000× *g* for 10 min (Universal 32 R, Hettich Group, Kirchlengern, Westphalia, Germany). After centrifugation, 20 µL of peptide stocks were diluted in PBS pH 7.20 at concentrations between 19.53–2500 µM (MC5 Micro Balance, Sartorius AG, Göttingen, Germany), and were dispensed in triplicate into 0.50 mL polypropylene vials. Eight 0.50 mL vials were filled with 20 µL of PBS pH 7.20 and eight with 20 µL of Triton X-100, serving as negative and positive hemolysis controls, respectively. After centrifugation, a 10% *v*/*v* hRBC solution was prepared in PBS pH 7.20. An amount of 230 µL of the 10% hRBC solution was added to each vial, and the mixture was mixed using inversion and incubated for 1 h at 100 rpm and 37 °C (Incubator 1000, Heidolph Instruments, Schwabach, Germany). After incubation, all vials were centrifuged at RT, 1000× *g* for 5 min (Universal 32 R, Hettich Group, Kirchlengern, Westphalia, Germany). Then, the supernatant was transferred to 96-well flat bottom culture plates without transferring of the RBC pellet, and hemoglobin release was measured at 420 nm (Multiskan SkyHigh, ThermoFisher Scientific, Waltham, MA, USA). All peptides were evaluated in three independent assays. The hemolysis percentage was calculated as follows:

$$Hemolysis\;\%=\left(\frac{{Abs}_{PEPTIDE}-{Median\;Abs}_{PBS}}{{Median\;Abs}_{TRITON}-{Median\;Abs}_{PBS}}\right)\times 100$$

Ten milliliters of fresh blood was mixed with 10 mL of PBS pH 7.20 in 50 mL conical tubes. Then, 10 mL of Ficoll^®^ density gradient media were placed in 50 mL conical tubes, and then the blood/PBS solution was gently poured over without disturbing the interface. The mixture was centrifuged at RT and 400× *g* for 30 min with the brake off (Universal 32 R, Hettich Group, Kirchlengern, Westphalia, Germany), and the upper plasma phase was removed and discarded. The leukocyte phase was gently removed from the conical tube, placed in a new 50 mL conical tube, and density gradient media was removed in two washings with 25 mL of PBS pH 7.20 at RT and 400× *g* for 10 min. Cells were then resuspended in 3 mL of RPMI media. Subsequently, 10 µL of this suspension was mixed with 90 µL of Trypan blue in a 0.50 mL polypropylene vial. The cell count and cell viability were determined in a Neubauer counting chamber. Then, cells were suspended to 3 × 10^5^ cells/mL, ensuring that cell viability remained above 98%. All peptides were diluted in RPMI media to final concentrations ranging between 1.56 and 200 µM. Subsequently, 20 µL of each peptide solution was added in triplicate into 96-well plates, along with 80 µL of fresh RPMI media. For negative and positive cytotoxicity controls, six wells received 20 µL of PBS and 30% *v*/*v* H_2_O_2_, respectively. Blank wells without cells were also included. Next, 100 µL of a cell suspension 3 × 10^5^ cells/mL was added to each well, and the plates were incubated for 24 h at 37 °C in a 5% CO_2_ atmosphere (Heratherm, ThermoFisher Scientific, Waltham, MA, USA). After 24 h, plates were centrifuged at RT and 1000× *g* for 10 min (Sorvall Legend XFR, ThermoFisher Scientific, Waltham, MA, USA). Then, 180 µL of the supernatant was collected, and 28 µL of a 4 mg/mL sterile MTT solution was added. The plates were then incubated for 4 h at 37 °C in a 5% CO_2_ atmosphere (Heratherm, ThermoFisher Scientific, Waltham, MA, USA). After the second incubation, 130 µL of DMSO was added to each well and plates were agitated at 50 rpm. The solubilized formazan was then measured at 570 nm using a Multiskan SkyHigh spectrophotometer (ThermoFisher Scientific, Waltham, MA, USA). All peptides were evaluated in three independent assays and the viability percentages were calculated as follows:

$$Viability\;\%=\left(1-\frac{{Abs}_{PEPTIDE}-{Median\;Abs}_{BLANK}}{{Median\;Abs}_{H_{2}O_{2}}-{Median\;Abs}_{BLANK}}\right)\times 100$$

### 4.7. Tables, Graphs, and Statistical Analysis

The raw data obtained from the Multiskan SkyHigh microplate reader (ThermoFisher Scientific, Waltham, MA, USA) were processed and tables were created using the software Microsoft Excel v.16.0+ (Microsoft Corporation, Redmond, WA, USA. Available at www.microsoft.com). Data from the initial screening were subjected to a one-way ANOVA followed by a Dunnett’s multiple comparison test to compare the peptides with the PBS negative control. A two-way ANOVA followed by a Dunnett’s multiple comparison test was performed for the initial growth inhibition kinetic assay, and then the FICI analysis, comparing PvAMP66 and PvAMP66/gentamicin to the PBS negative control, was performed. Graphs were created using the software GraphPad Prism v9.0.1 for Mac OS (GraphPad Software, La Jolla, CA, USA. Available at www.graphpad.com). Graph compositions were created using Microsoft PowerPoint v.16.0+ (Microsoft Corporation, Redmond, WA, USA. Available at www.microsoft.com).

## 5. Conclusions

The proposed methodology allowed the identification of 94 short, cationic, and amphiphilic peptides from the venom gland transcriptome of the *P. verdolaga*. Among the obtained peptides, nine showed antimicrobial activity against bacteria of the ESKAPE group in the mid-micromolar range. The peptide PvAMP66 displayed the most potent antimicrobial activity among the evaluated ATCC^®^ strains and the clinically isolated strain of *K. pneumoniae*. It is concluded that PvAMP66 is the first broad-spectrum AMP derived from the venom gland transcriptome of a spider of the *Pamphobeteus* genus. While the sequences obtained here are subject to potential further optimization, the observed additive interaction with gentamicin positions PvAMP66 as a prime candidate for more in-depth characterization.

## Figures and Tables

**Table 1:** Screening process of *P. verdolaga’s* AMPs.

Stimulus	*E. coli* ATCC^®^ 8739 (Inhibition %)	*E. coli* ATCC^®^ 25922 (Inhibition %)	*S. aureus* ATCC^®^ 6538 (Inhibition %)	*S. aureus* ATCC^®^ 29213 (Inhibition %)
PBS (C−)	0.00 ± 3.90	0.00 ± 5.72	0.07 ± 6.61	0.00 ± 4.34
BTM-P1 (C+)	85.52 ± 2.57 ****	96.89 ± 0.51 ****	71.10 ± 1.84 ****	94.12 ± 0.66 ****
PvAMP7	26.74 ± 0.68 ****	18.21 ± 2.54 ****	37.89 ± 1.50 ****	23.66 ± 3.34 **
PvAMP8	NA	NA	47.95 ± 3.62 ****	11.90 ± 8.93 ^ns^
PvAMP15	NA	NA	43.62 ± 10.56 ****	27.48 ± 29.69 ^ns^
PvAMP16	NA	NA	42.06 ± 4.29 ****	13.78 ± 6.48 ^ns^
PvAMP30	NA	NA	35.52 ± 7.29 ***	7.34 ± 10.33 ^ns^
PvAMP32	30.73 ± 5.21 ****	21.19 ± 3.82 ***	43.62 ± 4.90 *	33.17 ± 8.46 *
PvAMP66	88.81 ± 18.15 ****	73.59 ± 21.83 ****	139.72 ± 1.90 ****	98.25 ± 0.39 ****
PvAMP69	1.75 ± 9.78 ^ns^	19.08 ± 0.12 **	32.32 ± 7.00 ***	29.81 ± 3.31 ***
PvAMP81	8.57 ± 4.46 ^ns^	31.88 ± 2.18 ***	NA	NA
PvAMP82	64.01 ± 4.28 ****	74.94 ± 14.27 ****	43.03 ± 6.37 ****	43.44 ± 10.24 ****
PvAMP84	24.29 ± 6.74 ****	11.29 ± 3.72 ^ns^	NA	NA
PvAMP164	20.51 ± 4.31 ***	30.10 ± 3.90 ****	24.49 ± 10.18 **	12.25 ± 1.29 **
PvAMP169	13.60 ± 5.92 ^ns^	55.69 ± 4.65 ****	NA	NA
PvAMP172	64.78 ± 5.46 ****	47.43 ± 2.76 ****	NA	NA
PvAMP177	93.07 ± 7.11 ****	56.76 ± 3.64 ****	NA	NA
PvAMP179	NA	NA	50.42 ± 3.56 ****	10.43 ± 11.21 ^ns^
PvAMP183	48.26 ± 2.41 ****	64.66 ± 1.26 ****	47.36 ± 1.27 ****	4.71 ± 21.89 ^ns^

Note: C− = negative inhibition control. C+ = positive inhibition control. ns (not significant) = *p* > 0.05; * = *p* ≤ 0.05; ** = *p* ≤ 0.01; *** *p* ≤ 0.001; **** = *p* ≤ 0.0001. NA: No activity.

**Table 2:** MIC, MBC, and IC_50_ values against evaluated ESKAPE pathogens.

Peptide	Sequence	Activity Against	Parameter	µM	µg/mL
PvAMP7	SLWGMWR-NH_2_	*E. coli* ATCC^®^ 25922	MIC	200	23.38
PvAMP32	IIKKIWK-NH_2_	*E. coli* ATCC^®^ 25922	MIC	100	11.60
			MBC	100	11.60
			IC_50_	94.72 (77.98–118.05)	10.76
		*S. aureus* ATCC^®^ 29213	MIC	50	5.80
			IC_50_	107.66 (80.18–142.07)	12.49
		*P. aeruginosa* ATCC^®^ 9027	MIC	100	11.60
			MBC	100	11.60
			IC_50_	123.95 (93.17–160.2)	14.38
PvAMP66	WKKIKKFF-NH_2_	*E. coli* ATCC^®^ 25922	MIC	50	7.03
			MBC	50	7.03
			IC_50_	38.32 (32.03–46.14)	5.39
		*S. aureus* ATCC^®^ 29213	MIC	25	3.51
			MBC	50	7.03
			IC_50_	27.55 (21.07–27.55)	3.87
		*P. aeruginosa* ATCC^®^ 9027	MIC	12.5	177
			MBC	12.5	1.77
			IC_50_	12.67 (8.24–16.44)	1.78
		*K. pneumoniae* ATCC^®^ BAA-1705	MIC	100	14.06
			IC_50_	45.49 (35.22–60.76)	6.39
PvAMP69	YRARCVIYC-NH_2_	*E. coli* ATCC^®^ 25922	MIC	200	28.66
			MBC	200	28.66
PvAMP82	GRIFRLLRK-NH_2_	*E. coli* ATCC^®^ 25922	MIC	100	14.48
			MBC	100	14.48
		*P. aeruginosa* ATCC^®^ 9027	MIC	200	28.96
			MBC	200	28.96
PvAMP164	RSVLKAHCRICRRRG-NH_2_	*E. coli* ATCC^®^ 25922	MIC	200	45.28
			MBC	200	45.28
		*P. aeruginosa* ATCC^®^ 9027	MIC	100	22.64
			MBC	100	22.64
PvAMP172	CRKLCFRNRCLTYCRGR-NH_2_	*E. coli* ATCC^®^ 25922	MIC	50	13.43
			MBC	50	13.43
			IC_50_	42.91 (30.34–48.88)	11.52
		*S. aureus* ATCC^®^ 29213	MIC	100	26.86
			MBC	100	26.86
		*P. aeruginosa* ATCC^®^ 9027	MIC	50	13.43
			MBC	50	13.43
			IC_50_	48.43 (26.93–84.30)	13.01
PvAMP177	QCRKLCFRNRCLTYCRGR-NH_2_	*E. coli* ATCC^®^ 25922	MIC	25	7.11
			MBC	50	14.23
			IC_50_	25.32 (12.98–48.25)	6.64
		*S. aureus* ATCC^®^ 29213	IC_50_	137.91 (116.24–169.69)	37.82
		*P. aeruginosa* ATCC^®^ 9027	MIC	50	14.23
			MBC	50	14.23
			IC_50_	25.93 (14.02–47.01)	7.38
PvAMP183	QCRKLCFRNRCLTYCRGRG-NH_2_	*E. coli* ATCC^®^ 25922	MIC	25	6.98
			MBC	50	13.96
			IC_50_	26.89 (14.80–47.84)	7.51
		*P. aeruginosa* ATCC^®^ 9027	MIC	50	13.96
			MBC	100	27.92
			IC_50_	48.28 (26.02–88.63)	13.48

Note: In parentheses 95% confidence intervals. MIC: Minimum inhibitory Concentration. MBC: Minimum bactericidal concentration. IC_50_: Inhibitory concentration 50.

**Table 3:** MIC values against the MDR clinical isolate.

Stimulus	MIC (µM)	MIC (µg/mL)	IC_50_ (µM)	Classification at 10^6^ CFU/mL [30]
PvAMP66	25.00	3.51	15.80 (11.63–20.47)	NA
Gentamicin	50.00	9.31	33.38 (30.76–36.27)	Intermediate resistant
Chloramphenicol	100.00	4.04	56.23 (50.21–63.09)	Susceptible
Doxycycline	50.00	3.21	38.05 (28.95–51.87)	Susceptible

Note: In parentheses 95% confidence intervals. NA: Data not available. MIC: Minimum inhibitory concentration. IC_50_: Inhibitory concentration 50. CFU: Colony forming units.

**Table 4:** In vitro selectivity indexes for PvAMP66.

	Concentration (µM)	HC_50_/IC_50_	CC_50_/IC_50_
HC_50_	132.96 (118.82–151.21)	NA	NA
CC_50_	172.03 (124.44–ND)	NA	NA
IC_50_ *E. coli* ATCC^®^ 25922	38.32 (32.03–46.14)	3.47	4.49
IC_50_ *S. aureus* ATCC^®^ 29213	27.55 (21.07–27.55)	4.83	6.24
IC_50_ *P. aeruginosa* ATCC^®^ 9027	12.67 (8.24–16.44)	10.49	13.58
IC_50_ *K. pneumoniae* ATCC^®^ BAA-1705	45.49 (35.22–60.76)	2.92	3.78
IC_50_ MDR *K. pneumoniae*	15.80 (11.63–20.47)	8.42	10.89

Note: In parentheses 95% confidence intervals. NA: Not applicable. ND: Above evaluated concentration range.

## Data Availability

All the assemblies associated to the *P. verdolaga’s* venom gland transcriptome are deposited at DDBJ/EMBL/GenBank under the accession GIUY00000000. All the hypothetical mature toxins associated to the venom gland transcriptome are deposited at the EMBL-EBI European Nucleotide Archive under accession numbers OX043992 to OX044319. The data presented in this study is available in Supplementary Materials. Further required data is available on request from the corresponding author.

## Supplementary Materials

The following supporting information can be downloaded at: https://www.mdpi.com/article/10.3390/antibiotics13010006/s1. Table S1. Custom database (cDB) of arthropod antimicrobial peptides. Table S2. List of *P. verdolaga’s* hypothetical AMPs. Table S3. LC/MS analysis of *P. verdolaga’s* AMPs. Table S4. IC_50_ values for isobologram analysis. Figure S1. RP-HPLC analysis of *P. verdolaga’s* AMPs. Figure S2. Dose-response curves of *P. verdolaga’s* AMPs against *E. coli* ATCC^®^ 25922. Figure S3. Dose-response curves of *P. verdolaga’s* AMPs against *S. aureus* ATCC^®^ 29213. Figure S4. Dose-response curves of *P. verdolaga’s* AMPs against *E. faecalis* ATCC^®^ 49532. Figure S5. Dose-response curves of *P. verdolaga’s* AMPs against *P. aeruginosa* ATCC^®^ 9027. Figure S6. Dose-response curves of *P. verdolaga’s* AMPs against *K. pneumoniae* ATCC^®^ BAA-1705. Figure S7. Dose-response curves of MDR *K. pneumoniae* against antibiotics. Figure S8. Dose-response curves of *P. verdolaga’s* AMPs against MDR *K. pneumoniae*. Figure S9. SEM analysis. Figure S10. Dose-response curves of the isobologram analysis.
