# Supplementary material for: Short Antimicrobial Peptide Derived from the Venom Gland Transcriptome of Pamphobeteus verdolaga Increases Gentamicin Susceptibility of Multidrug-Resistant Klebsiella pneumoniae
Source: Antibiotics (Basel). 2023 Dec 20;13(1):6. doi: 10.3390/antibiotics13010006 (PMC10812672; doi:10.3390/antibiotics13010006)
Supplement: Supplementary file 1 [file antibiotics-13-00006-s001.zip › antibiotics-2685173-SI.pdf]

Supplementary Material

Short Antimicrobial Peptide Derived from the Venom Gland Transcriptome of *Pamphobeteus verdolaga* Increases Gentamicin Susceptibility of Multidrug-Resistant *Klebsiella pneumoniae*

Cristian Salinas-Restrepo [1], Ana María Naranjo-Duran [1], Juan Quintana [2], Julio Bueno [3], Fanny Guzman [4], Lina M. Hoyos Palacio [5] and Cesar Segura [6,*]

[1] Grupo Toxinología, Alternativas Terapéuticas y Alimentarias, Facultad de Ciencias Farmacéuticas y Alimentarias, Universidad de Antioquia, Medellín 050012, Colombia; cristian.salinas@udea.edu.co (C.S.-R.); amaria.naranjo@udea.edu.co (A.M.N.-D.)
[2] Facultad de Medicina, Universidad Cooperativa de Colombia, Medellín 050012, Colombia; juan.quintanac@ucc.edu.co
[3] Grupo Reproducción, Facultad de Medicina, Universidad de Antioquia, Medellín 050012, Colombia; julio.bueno@udea.edu.co
[4] Núcleo Biotecnología Curauma (NBC), Pontificia Universidad Católica de Valparaíso, Valparaíso 3100000, Chile; fanny.guzman@pucv.cl
[5] Escuela de Ciencias de la Salud, Grupo de Investigación Biología de Sistemas, Universidad Pontificia Bolivariana, Medellín 050031, Colombia; lina.hoyos@upb.edu.co
[6] Grupo Malaria, Facultad de Medicina, Universidad de Antioquia, Medellín 050012, Colombia
* Correspondence: cesar.segura@udea.edu.co

Table S1. Custom database (cDB) of arthropod antimicrobial peptides.

Peptide	Species	Sequence	# aa	Charge (+)	MIC *E. coli* (μM)	MIC *S. aureus* (μM)
AaeAP1	*Androctonus aeneas*	FLFSLIPSVIAGLVSAIRN	19	1		11.90
AaeAP1 [S4.8.15K; A11K. N19K]	*Androctonus aeneas*	FLFKLIPKVIKGLVKAIRK	19	6	10.85	8.14
AaeAP2	*Androctonus aeneas*	FLFSLIPSAIAGLVSAIRN	19	1		8.04
AaeAP2 [S4.8.15K; A11K. N19K]	*Androctonus aeneas*	FLFKLIPKAIKGLVKAIRK	19	6	10.99	8.24
AamAP1 [S4K.H8K.G11.12 K.A16K]	*Androctonus amoreuxi*	FLFKLIPKAIKKLISKFK	18	6	7.50	5.00
AamAP1 [H8K]	*Androctonus amoreuxi*	FLFSLIPKAIGGLISAFK	18	2	5.00	3.00
AcrAP1	*Androctonus crassicauda*	FLFSLIPHAISGLISAFK	18	1		8.00
AcrAP1a	*Androctonus crassicauda*	FLFKLIPKAIKGLIKAFK	18	6	4	8
AcrAP1a [S4K.H8K.S11K.S 15K]	*Androctonus crassicauda*	FLFKLIPKAIKGLIKAFK	18	5	8.00	4.00
AcrAP2	*Androctonus crassicauda*	FLFSLIPNAISGLLSAFK	18	1		8.00

AcrAP2 [S4K.N8K.S11K.S15K]	*Androctonus crassicauda*	FLFKLIPKAIKGLLKAFK	18	5	8.00	4.00
Agelaia-CP	*Agelaia pallipes*	ILGTILGLLKGL	12	1		12.00
Androctonin	*Androctonus australis*	RSVCRQIKICRRRGGCYYKCTNRPY	25	8	12.00	
Apidaecin-1A	*Apis mellifera*	GNNRPVYIPQPRPPHPR	17	3	11.71	
Apidaecin-1B	*Apis mellifera*	GNNRPVYIPQPRPPHPRL	18	3	0.32	
Apidaecin-2	*Apis mellifera*	GNNRPIYIPQPRPPHPRL	18	3	0.09	
Bicarinalin	*Tetramorium bicarinatum*	KIKIPWGKVKDFLVGGMKAV	20	4		10.45
BmKn1	*Mesobuthus martensii*	FIGAVAGLLSKIF	13	1		14.97
Bmkn2	*Mesobuthus martensii*	FIGAIARLLSKIFGKR	16	4	1.50	0.60
BmKn2OR	*Mesobuthus martensii*	FIGAIARLLSKIF	13	2		5.49
cGomesin	*Acanthoscurria gomesiana*	GCRRLCYKQRCVTYCRGR	18	6	2.03	
Codesane	*Colletes daviesanus*	GMASLLAKVLPHVVKLIK	18	3	3.70	3.70
Css54	*Centruroides suffusus suffusus*	FFGSLLSLGSKLLPSVFKLFQRKKE	25	4	4.35	4.35
Ctriporin	*Chaerilus tricostatus*	FLWGLIPGAISAVTSLIKK	19	2		4.34
Cupiennin-1d	*Cupiennius salei*	GFGSLFKFLAKKVAKTVAKQAAKQGAKYVANKHME	35	7	0.12	0.94
Cupiennin-2a	*Cupiennius salei*	GFGTILKALAKIAGKVVKKLATKPGATYMLKENLK	35	7	0.16	0.16
Defensin-1	*Centruroides limpidus limpidus*	ACQFWSCNSSCISRGYRQGYCWGIQYKYCQCQ	32	3	13.08	13.08
Delta-Myrtoxin-Mp1a	*Myrmecia pilosula*	IDWKKVDWKKVSKKTCKVMLKACKFLG	27	7		4.80
Dominulin-A	*Polistes dominula*	INWKKIAEVGGKILSSL	17	2	4.31	
Dominulin-B	*Polistes dominula*	INWKKIAEIGKQVLSAL	17	2	4.19	
Eumenine mastoparan-AF	*Anterhynchium flavomarginatum*	INLLKIAKGIIKSL	14	3	13.12	3.28
Eumenitin	*Eumenes rubronotatus*	LNLKGIFKKVASLLT	15	3	6.00	
Gomesin	*Acanthoscurria gomesiana*	CRRLCYKQRCVTYCRGR	17	6	0.80	12.10
Gomesin (4-18) [Q9p]	*Acanthoscurria gomesiana*	RLTYKPRTVTYTRGR	15	5	10.24	
Gomesin c(1-18)[Gln1]	*Acanthoscurria gomesiana*	QCRRLCYKQRCVTYCRGR	18	6	0.48	1.44
H/V-peptide (1-11)(19-25)	*Hadrurus aztecus*	GILKTIKSIASKLKRKAK	18	7	9.37	
H/V-peptide (1-28)	*Vaejovis mexicanus smithi*	GILKTIKSIASKVANTVQKLKRKAKNAV	28	8	1.20	
HAL-1	*Halictus sexcinctus*	GMWSKILGHLIR	12	2	3.80	7.70

HAL-1/10	*Halictus sexcinctus*	GMWKKILGKLIR	12	4	2.30	15
HAL-1/15	*Halictus sexcinctus*	GMWSKLLGHLLR	12	2	3.00	7.70
HAL-1/18	*Halictus sexcinctus*	GMWSKILKHLIR	12	3	2.30	3.70
HAL-1/19	*Halictus sexcinctus*	GKWKKILGHLIR	12	4	2.70	
HAL-1/20	*Halictus sexcinctus*	GKWSKILGKLIR	12	4	2.30	
HAL-1/21	*Halictus sexcinctus*	GKWKKILGKLIR	12	5	1.80	
HAL-1/26	*Halictus sexcinctus*	GMWSKILGHLI	11	1	7.00	
HAL-1/5	*Halictus sexcinctus*	GMWKKILGHLIR	12	3	1.80	8.40
HAL-1/6	*Halictus sexcinctus*	GMWSKILGHLIK	12	2	7.20	
HAL-1/9	*Halictus sexcinctus*	GMWSKILGKLIR	12	3	3.70	9.20
HAL-2	*Halictus sexcinctus*	GKWMSLLKHILK	12	3	2.50	8.10
HAL-2/1	*Halictus sexcinctus*	GKWKSLLKHILK	12	4	3.70	
HAL-2/10	*Halictus sexcinctus*	GKWWSLLKHILK	12	3	1.60	4.70
HAL-2/11	*Halictus sexcinctus*	GKWLSLLKHILK	12	3	1.80	5.50
HAL-2/12	*Halictus sexcinctus*	GKWMHLLKHILK	12	3	3.70	12.50
HAL-2/13	*Halictus sexcinctus*	GKWMTLLKHILK	12	3	2.70	5.30
HAL-2/14	*Halictus sexcinctus*	GKWMSLLKQILK	12	3	2.50	10.80
HAL-2/16	*Halictus sexcinctus*	SKWMSLLKHILK	12	3	2.30	9.20
HAL-2/17	*Halictus sexcinctus*	HKWMSLLKHILK	12	3	3.50	
HAL-2/2	*Halictus sexcinctus*	GKWMKLLKHILK	12	4	2.50	4.50
HAL-2/20	*Halictus sexcinctus*	GKWMSLWKHILK	12	3	8.00	
HAL-2/21	*Halictus sexcinctus*	GKWMSWLKHILK	12	3	3.90	10.10
HAL-2/24	*Halictus sexcinctus*	GKFMSLLKHILK	12	3	5.80	
HAL-2/4	*Halictus sexcinctus*	GKWMSLLKKILK	12	4	2.70	6.30
HAL-2/5	*Halictus sexcinctus*	KKWMSLLKHILK	12	4	6.30	
HAL-2/6	*Halictus sexcinctus*	GKWMSFLKHILK	12	3	4.00	6.40
HAL-2/9	*Halictus sexcinctus*	GKWQSLLKHILK	12	3	8.00	
Heterin 2	*Heterometrus spinifer*	FWGALAKGALKLIPSLVSSFTKKD	24	3		5.60
Hp1090	*Heterometrus petersii*	FKAIWSGIKSLF	12	2	15	
Hp1404	*Heterometrus petersii*	GILGKLWEGVKSIF	14	1		8.75

HYL	*Hylaeus signatus*	GIMSSLMKKLAAHIAK	16	3	15	
Im-5	*Isometrus maculatus*	FLGSLFSIGSKLLPGVIKLFQRKKQ	25	5	10.00	3.75
IsCT	*Opisthacanthus madagascariensis*	ILGKIWEGIKSLF	13	1	4.00	1.25
IsCT [EK7. GP8. SK11]	*Opisthacanthus madagascariensis*	ILGKIWKPIKKLF	13	4	2.00	1.17
IsCT [EK7]	*Opisthacanthus madagascariensis*	ILGKIWKGIKSLF	13	3	2.00	1.00
IsCT [WL6. SK11]	*Opisthacanthus madagascariensis*	ILGKILKGIKKLF	13	4	2.00	2.00
IsCT [WL6]	*Opisthacanthus madagascariensis*	ILGKILEGIKSLF	13	1		4.00
IsCT precursor	*Opisthacanthus madagascariensis*	ILGKIWKIKKLF	12	4	2.00	2.00
IsCT-P	*Opisthacanthus madagascariensis*	ILKKIWKPIKKLF	13	5	2.00	1.00
Ixosin	*Ixodes sinensis*	GLHKVMREVLGYERNSYKKFFLR	23	4	10.45	1.29
Jellein-1	*Apis mellifera*	PFKLSLHL	8	1	2.62	10.48
Jellein-2	*Apis mellifera*	TPFKLSLHL	9	1	14.21	
Lacrain	*Scolopendra viridicornis*	RYPAVGYT	8	1	5.80	
Lasiocepsin	*Lasioglossum laticeps*	GLPRKILCAIAKKKGKCKGPLKLVCKC	27	9	3.00	
Lasioglossin LL-I	*Lasioglossum laticeps*	VNWKKVLGKIIKVAK	15	5	1.70	14.3
Lasioglossin LL-II	*Lasioglossum laticeps*	VNWKKILGKIIKVAK	15	5	1.40	9.00
Lasioglossin LL-III	*Lasioglossum laticeps*	VNWKKILGKIIKVVK	15	5	1.40	3.90
Latarcin-1	*Lachesana tarabaevi*	SMWSGMWRRKLKKLRNALKKKLKGEK	26	10	0.85	
Latarcin-2a	*Lachesana tarabaevi*	GLFGKLIKKFGRKAISYAVKKARGKN	26	10	0.7	
Latarcin-3a	*Lachesana tarabaevi*	SWKSMAKKLKEYMEKLKQRA	21	5	4.25	
Latarcin-4a	*Lachesana tarabaevi*	GLKDKFKSMGEKLKQYIQTWKAKF	25	5	3.85	
Latarcin-4b	*Lachesana tarabaevi*	SLKDKVKSMGEKLKQYIQTWKAKF	24	5	4.40	
Latarcin-5	*Lachesana tarabaevi*	GFFGKMKEYFKKFGASFKRRFANLKKRL	28	9	0.60	0.70
Longicin (53-73)	*Haemaphysalis longicornis*	SIGRRGGYCAGIIKQTCTCYR	21	4	2.00	2.00
Lycocitin-1	*Lycosa singoriensis*	GKLQAFLAKMKEIAAQTL	19	2	4.80	
Lycocitin-2	*Lycosa singoriensis*	GRLQAFLAKMKEIAAQTL	18	2	4.70	
Lycosin-I	*Lycosa singoriensis*	RKGWFKAMKSIAKFIAKEKLKEHL	24	6		7.80

Lycosin-II	*Lycosa singoriensis*	VWLSALKFIGKHLAKHQLSKL	21	4	12.50	3.10
M-ctenitoxin-Cs1a	*Cupiennius salei*	GFGALFKFLAKKVAKTVAKQAAKQGAKYVVNKQME	35	7	0.47	0.47
M-lycotoxin-Hc1a	*Hogna carolinensis*	IWLTALKFLGKHAAKHLAKQQLSKL	26	5	15	
M-theraphotoxin-Gr1a	*Grammostola rosea*	GCLEFWWKCNPNDDKCCRPKLKCSKLFKLCNFSF	34	4	12.00	3.00
Macropin 1	*Macropis fulvipes*	GFGMALKLLKKVL	13	3	3.00	3.70
Marmelittin	*Mesobuthus martensii*	FLFSLIPSAISGLISAFKGRR	21	3	6.21	6.48
Mastoparan B	*Vespa basalis*	LKLKSIVSWAKKVL	14	4		8.89
Mastoparan MP	*Mischocyttarus phthisicus*	INWLKLGKKMMSAL	14	3		9.00
Mastoparan PDD-A	*Polistes dorsalis dorsalis*	INWKKIFEKVKNLV	14	3	5.62	11.80
Mastoparan PMM	*Polistes major major*	INWKKIASIGKEVLKAL	17	3	4.93	
Mastoparan-A	*Vespa analis*	IKWKAILDAVKKVI	14	3	4.92	
Mastoparan-AF	*Vespa magnifica*	INLKAIAALAKKLF	14	3	3.96	
Mastoparan-L	*Vespa tropica*	INLKALAALAKKIL	14	3		14.6
Mastoparan-like peptide 12b	*Vespa magnifica*	INWKGIAAMKKLL	13	3	10.09	2.49
Mastoparan-like peptide 12c	*Vespa magnifica*	INLKAIAALAKKLLG	15	3	3.90	1.95
Mastoparan-like peptide 12d	*Vespa magnifica*	INLKAIAAMAKKLL	14	3	2.00	1.00
Mastoparan-M	*Vespa magnifica*	INLKAIAALAKKLL	14	3	5.41	
Mastoparan-T	*Vespa tropica*	INLKAIAAFAKKLL	14	3	5.28	
Mastoparan-V	*Vespa magnifica*	INWKGIAAMAKKLL	14	3	5.13	
Mastoparan-VT2	*Vespa tropica*	NLKAIAALAKKLL	13	3	10.06	5.49
Mastoparan-VT3	*Vespa tropica*	INLKAITALAKKLL	14	3		3.86
Mastoparan-VT7	*Vespa tropica*	INLKAIAALARNY	13	2		13.98
Melectin	*Melecta albifrons*	GFLSILKKVLPKVMAHMK	18	4	10.37	12.80
Melittin	*Apis florea*	GIGAILKVLATGLPTLISWIKNKRKQ	26	5	0.60	0.60
Melittin	*Apis mellifera*	GIGAVLKVLTTGLPALISWIKRKRQQ	26	5	2.72	2.78
Meucin-13	*Mesobuthus eupeus*	IFGAIAGLLKNIF	13	1	7.90	8.89
Meucin-18	*Mesobuthus eupeus*	FFGHLFKLATKIIPSLFQ	18	2	12.56	4.29
Midgut defensin G2	*Haemaphysalis longicornis*	ACHAHCQSVGRRGGYCGNFRMTCYCY	26	3	5.94	5.09
MP-VB1	*Vespa bicolor*	INMKASAAVAKKLL	14	3		2.15
Mucroporin	*Lychas mucronatus*	LFGLIPSLIGGLVSAFK	17	1		14.43
OcyC1	*Urodacus yaschenkoi*	ILSAIWSGIKSLF	13	1		6.50
OsDef2 (16-37)	*Ornithodoros savignyi*	KGIRGYKGGYCKGAFKQTCKCY	22	6		6.10

OsDef2 (16-37)[C26.34.36Del]	*Ornithodoros savignyi*	KGIRGYKGGYKGAFKQTKY	19	6		6.98
Osmin	*Osmia rufa*	GFLSALKKYLPIVLKHV	17	3	1.56	
OxTx1	*Oxyopes takobius*	KFKWGKLFSTAKKLYKKGKKLSKNKNFKKALKFGK	35	15	0.19	0.93
Oxyopinin-4a	*Oxyopes takobius*	GIRCPKSWKCKAFKQRVLKRLLAMLRQHAF	31	9	0.50	10.00
Pandinin-2	*Pandinus imperator*	FWGALAKGALKLIPSLFSSFSKKD	24	3		12.34
Pandinin-2 [P14G]	*Pandinus imperator*	FWGALAKGALKLIGSLFSSFSKKD	24	3	12.50	12.50
Pantinin-1	*Pandinus imperator*	GILGKLWEGFKSIV	14	1		11.00
Pantinin-3	*Pandinus imperator*	FLSTIWNGIKSLL	13	1		14
Parapolybia Mastoparan	*Parapolybia indica*	INWKKMAATALKMI	14	3	2.41	1.79
Pepcon	*Androctonus amoreuxi*	FLFSLIPSAIGGLISAFK	18	1		5.00
Peptide BmKb1	*Mesobuthus martensii*	FLFSLIPSAISGLISAFK	18	1		7.27
Pilosulin-1 (1-20)	*Myrmecia pilosula*	GLGSVFGRLARILGRVIPKV	20	4	3.33	2.50
Pilosulin-2	*Myrmecia pilosula*	GLLSKFGRLARKLARVIPKV	20	6	1.33	4.00
PNG-1	*Panurgus calcaratus*	LNWGAILKHIIK	12	2	3.70	10.60
PNG-1/1	*Panurgus calcaratus*	NLWAGILKHIIK	12	2	12.50	
PNG-1/12	*Panurgus calcaratus*	KNWGKILKHIIK	12	4	11.30	
PNG-1/13	*Panurgus calcaratus*	KNWKAILKHIIK	12	4	7.80	
PNG-1/17	*Panurgus calcaratus*	LNWGAFLKHFFK	12	2	5.00	
PNG-1/19	*Panurgus calcaratus*	LNWGALLKHLLK	12	2	3.80	6.70
PNG-1/22	*Panurgus calcaratus*	LNWGAWLKHWWK	12	2	7.20	
PNG-1/3	*Panurgus calcaratus*	LKWGAILKHIIK	12	3	3.70	11.70
PNG-1/5	*Panurgus calcaratus*	LNWKAILKHIIK	12	3	2.30	5.30
PNG-1/6	*Panurgus calcaratus*	LNWGKILKHIIK	12	3	4.00	8.70
PNG-1/9	*Panurgus calcaratus*	LNWGAILKKIIK	12	3	4.30	7.30
Polybia-CP	*Polybia paulista*	ILGTILGLLKSL	12	1		4.00
Polybia-MP-II	*Polybia paulista*	INWLKLGKMVIDAL	14	1	4.96	2.36
Polyphemusin-2	*Limulus polyphemus*	RRWCFRVCYKGFCYRKCR	18	8	3.87	5.14
Polyphemusin-1	*Limulus polyphemus*	RRWCFRVCYRGFCYRKCR	18	8	0.9	0.3
Ponericin-G1	*Pachycondyla goeldii*	GWKDWAKKAGGWLKKKGPGMAKAALKAAMQ	30	7		8.00
Ponericin-W1	*Pachycondyla goeldii*	WLGSALKIGAKLLPSVVGLFKKKKQ	25	6		8.00

Ponericin-W4	*Pachycondyla goeldii*	GIWGTALKWGVKLLPKLVGMAQTKKQ	26	5	7.25	12.50
Protonectarina Mastoparan	*Protonectarina sylveirae*	INWKALLDAAKKVL	14	2	4.93	2.46
Salivary glands defensin S2	*Haemaphysalis longicornis*	NCIQQCVSKGAQGGYCTNEKCTCY	24	1	6.73	5.76
Scolopendin 2	*Scolopendra subspinipes mutilans*	AGLQFPVGRIGRLLRK	16	4	6.30	
Scolopin 1	*Scolopendra mutilans*	FLPKMSTKLRVPYRRGTKDYH	21	5	5.30	1.20
Scolopin 2	*Scolopendra mutilans*	GILKKFMLHRGTKVYKMRTLSKRSH	25	8	5.00	0.25
Smp24 - GVG	*Scorpio maurus palmatus*	IWSFLIKAATKLLGVGSLFGGGKKDS	26	3		5.94
Smp24-T	*Scorpio maurus palmatus*	IWSFLIKAATKLLPSLFGG	19	2		7.76
Tachyplesin-3	*Tachypleus gigas*	KWCFRVCYRGICYRKCR	17	7	7.1	1.8
Tachyplesin-2	*Tachypleus tridentatus*	RWCFRVCYRGICYRKCR	17	7	4.1	1
Tachyplesin-1	*Tachypleus gigas*	KWCFRVCYRGICYRRCR	17	7	1.8	1.3
Toxin LyeTx 1	*Lycosa erythrognatha*	IWLTALKFLGKNLGKHLAKQQLAKL	25	5	6.66	4.65
TsAP2	*Tityus serrulatus*	FLGMIPGLIGGLISAFK	17	1		7.50
Um3	*Urodacus manicatus*	GFWGKLWEGVKSAI	14	1	15	15
Um4	*Urodacus manicatus*	FFSALLSGIKSLF	13	1	8.00	15.00
Um5	*Urodacus manicatus*	IFKAIWSGIKSLF	13	2	15.00	
Uy192	*Urodacus yaschenkoi*	FLSTIWNGIKGLL	13	1	15.00	
Uy192 [G11S.L13F]	*Urodacus yaschenkoi*	FLSTIWNGIKSLF	13	1		8.00
Uy234 [Ins 5.9.12.16K]	*Vaejovis punctatus*	FPFLKLSLKIPKSAIKSAIKRL	22	6	8.00	
UyCT1	*Urodacus yaschenkoi*	GFWGKLWEGVKNAI	14	1		6.75
UyCT1 - 3K	*Urodacus yaschenkoi*	GFWGKLWEGVKNAIKKK	17	4	4.00	
UyCT1 [W7L.N12K]	*Urodacus yaschenkoi*	GFWGKLLEGVKKAI	14	2	15.00	
UyCT3 [L2F.S3G]	*Urodacus yaschenkoi*	IFGAIWSGIKSLF	13	1	8.00	
UyCT5	*Urodacus yaschenkoi*	IWSAIWSGIKGLL	13	1		2.75
VCP 5f	*Vespa magnifica*	FLPIPRPILLGLL	13	1		6.84
VCP 5g	*Vespa magnifica*	FLIIRRPIVLGLL	13	2		6.57
VCP-VT2	*Vespa tropica*	FLPIIGKLLSG	11	1	8.64	5.04
Ves-CP-M	*Vespa magnifica*	FLPIIGKLLSGLL	13	1		8.00
Ves-CP-X	*Vespa magnifica*	FLPIIAKLLGGLL	13	1		3.66
VESP-VB1	*Vespa bicolor*	FMPIIGRLMSGSL	13	1	7.91	1.56

VmCT1 [N7K]	*Vaejovis mexicanus smithi*	FLGALWKVAKSVF	13	2	1.56	6.56
VmCT1 [A9W]	*Vaejovis mexicanus smithi*	FLGALWNVWKSVF	13	1	1.56	1.56
VmCT1 [G3K]	*Vaejovis mexicanus smithi*	FLKALWNVAKSVF	13	2	1.56	3.12
VmCT1 [S11K]	*Vaejovis mexicanus smithi*	FLGALWNVAKKVF	13	2	1.56	1.56
VmCT2	*Vaejovis mexicanus smithi*	FLSTLWNAAKSIF	13	1		10.00
VpAmp1.0	*Vaejovis punctatus*	LPFFLLSLIPSAISAIKKI	19	2		2.50
VpAmp1.1	*Vaejovis punctatus*	FFLLSLIPSAISAIKKI	17	2		5.00
VpAmp2.0	*Vaejovis punctatus*	FWGFLGKLAMKAVPSLIGGNKSSSK	25	4		10.00
VpAmp2.1	*Vaejovis punctatus*	FWGFLGKLAMKAVPSLIGGNKK	22	4		5.00
Xac-2	*Xylocopa appendiculata*	GFVALLKKLPLILKHLP	17	3	3.12	3.12

Table S2. List of *P. verdolaga's* hypothetical AMPs.

Id.	Candidate	A	B	C	Homologous to	D	Contig id
PvAMP0	CFYKCTN	7	2	1.25	Androctonin	85.71	Pv_tr_17126.t1
PvAMP1	CRPKLRC	7	4	1.22	M-theraphotoxin-Gr1a	85.71	Pv_sp_5188.t1
PvAMP2	QRAVTYC†	7	2	1.25	cGomesin	85.71	Pv_tr_14213.t1
PvAMP3	IQTIKAK†	7	3	1.23	M-zodatoxin-Lt4a	85.71	Pv_sp_1494.t1
PvAMP5	KQKCVTY	7	3	1.95	Gomesin	85.71	Pv_so_5570.t1
PvAMP6	YKCTYRP	7	3	2.32	Androctonin	85.71	Pv_tr_16614.t1
PvAMP7	SLWGMWR	7	2	2.33	M-zodatoxin-Lt1a	75	Pv_sp_3683.t1
PvAMP8	SLFKFLA	7	2	0.52	Cupiennin-1d	100	Pv_tr_9174.t1
PvAMP9	NSLRKVQ	7	3	1.05	Lt4a	85.71	Pv_tr_16907.t1
PvAMP11	INRKLLE	7	2	1.06	Lt4b	85.71	Pv_sp_4704.t1
PvAMP12	AKMREIA	7	2	1.06	Lycocitin 1	85.71	Pv_so_4563.t1
PvAMP13	DKFKAMG	7	2	1.05	M-zodatoxin-Lt4a	85.71	Pv_tr_17094.t1
PvAMP14	KEKLKEH	7	2	2.14	Lycosin-I	100	Pv_so_3998.t1
PvAMP15	FLGKNLG	7	2	0.52	Lycosin-I	100	Pv_sp_6402.t1
PvAMP16	LLSKIFG†	7	2	0.52	Bmkn2	100	Pv_tr_2228.t1
PvAMP17	GMISAFK	7	2	0.52	TsAP2	85.71	Pv_sp_6035.t1
PvAMP18	KIIPNLF	7	2	0.52	Meucin-18	85.71	Pv_sp_2233.t1
PvAMP19	FVFKLIP	7	2	0.52	AcrAP2a	85.71	Pv_tr_2209.t1
PvAMP20	IKKLISK	7	4	1.57	AamAP1	100	Pv_sp_1634.t1
PvAMP21	LVKAVRK	7	4	1.4	AaeAP1 [S4.8.15K; A11K. N19K]	85.71	Pv_sp_6551.t1
PvAMP22	LLPSIFK	7	2	0.52	Css54	85.71	Pv_sp_192.t1
PvAMP23	RIGRILR	7	4	1.05	Scolopendin 2	85.71	Pv_sp_225.t1
PvAMP24	VTYTRDR	7	2	1.42	Gomesin (4-18) [Q9p]	85.71	Pv_tr_1752.t1
PvAMP25	IWKIKKL	7	4	2.56	IsCT precursor	100	Pv_sp_743.t1
PvAMP26	VLGKIWK	7	3	2.04	IsCT precursor	85.71	Pv_sp_13985.t1
PvAMP27	GLLKNVF	7	2	0.52	Meucin-13	85.71	Pv_sp_7193.t1
PvAMP28	KLLSKIF	7	3	1.05	Bmkn2	100	Pv_sp_556.t1
PvAMP29	IFKSLWS	7	2	1.51	Um5	71.43	Pv_sp_550.t1
PvAMP30	LLTGIKS	7	2	0.52	Um4	85.71	Pv_sp_8010.t1
PvAMP31	ILGKILR	7	3	0.87	IsCT [WL6. SK11]	100	Pv_so_7688.t1
PvAMP32	IIKKIWK	7	4	2.56	IsCT-P	85.71	Pv_so_7981.t1
PvAMP33	RVAKSVF	7	3	0.87	VmCT1	100	Pv_tr_5074.t1
PvAMP34	LWNVVKK	7	3	2.04	VmCT1 [S11K]	85.71	Pv_tr_5073.t1
PvAMP35	NIWKSVY	7	2	2.24	VmCT1 [A9W]	71.43	Pv_sp_1355.t1
PvAMP36	VCYKGHCY†	8	2	1.9	Polyphemusin-2	87.5	Pv_tr_15238.t1
PvAMP37	KILKHVIK†	8	4	1.56	PNG-1/6	87.5	Pv_sp_2023.t1
PvAMP38	YIQTWHTK†	8	2	2.3	M-zodatoxin-Lt4b	100	Pv_tr_7193.t1
PvAMP39	RGYKGGHC†	8	3	1.58	OsDef2 (16-37)	87.5	Pv_sp_11752.t1
PvAMP41	KCCEGNRR	8	3	1.23	κ-theraphotoxin-Gr4a	72.73	Pv_sp_3172.t1
PvAMP42	FADLKKRL	8	3	1.22	M-zodatoxin-Lt5a	87.5	Pv_tr_11159.t1
PvAMP43	VAKTVTKQ	8	3	1.07	M-ctenitoxin-Cs1a	87.5	Pv_sp_67.t1
PvAMP45	RKLMEINL	8	2	0.92	Lt4a	75	Pv_tr_7666.t1
PvAMP46	DRKLMQII	8	2	0.92	Lt4a	62.5	Pv_sp_3014.t1
PvAMP47	LTKMKEIA	8	2	1.08	Lycocitin 1	87.5	Pv_sp_2708.t1
PvAMP48	KAMKAIAK	8	4	1.38	Lycosin-I	87.5	Pv_tr_4264.t1
PvAMP49	KDKLKEHM	8	2	1.72	Lycosin-I	75	Pv_tr_3957.t1
PvAMP50	FKAMKGVA	8	3	0.92	Lycosin-I	75	Pv_tr_1918.t1
PvAMP51	LLSRIFGK	8	3	0.76	Bmkn2	87.5	Pv_tr_9659.t1
PvAMP52	VRNAIRKK	8	5	1.53	UyCT1 - 3K	75	Pv_tr_9657.t1
PvAMP53	KLAAKIIP	8	3	0.92	Meucin-18	87.5	Pv_sp_1006.t1
PvAMP54	SGMISAFK	8	2	0.46	Marmelittin	87.5	Pv_sp_6035.t1
PvAMP56	KGLLKDFK	8	3	1.38	AcrAP2a	87.5	Pv_sp_2642.t1
PvAMP57	PGAIKKLI	8	3	0.92	AamAP1	87.5	Pv_sp_2640.t1
PvAMP58	IPKVVKGM	8	3	0.92	AaeAP1 [S4.8.15K; A11K. N19K]	75	Pv_sp_5515.t1
PvAMP59	FPVGRVAR	8	3	0.61	Scolopendin 2	75	Pv_sp_6943.t1
PvAMP60	GAIAKLLS	8	2	0.46	Bmkn2	87.5	Pv_tr_6401.t1

PvAMP61	LSTRWNGI	8	2	1.17	Uy192	87.5	Pv_tr_13580.t1
PvAMP62	LGKILDIK	8	2	0.92	IsCT [WL6]	77.78	Pv_tr_13579.t1
PvAMP63	ALNVAKSV	8	2	0.46	VmCT1 [G3K]	88.98	Pv_tr_7191.t1
PvAMP64	KALWNILK	8	3	1.78	VmCT1 [G3K]	75	Pv_so_8363.t1
PvAMP65	ALWNILKK	8	3	1.78	VmCT1 [S11K]	75	Pv_so_8363.t1
PvAMP66	WKKIKKFF	8	5	2.7	IsCT [EK7. GP8. SK11]	75	Pv_tr_5073.t1
PvAMP67	KLLNGVKK	8	4	1.38	UyCT1 [W7L.N12K]	87.5	Pv_sp_1577.t1
PvAMP68	LEGVKRAV	8	2	0.92	UyCT1 [W7L.N12K]	75	Pv_sp_5735.t1
PvAMP69	YRARCVIYC	9	3	1.67	Gomesin c(1-18)[Gln1]	66.67	Pv_tr_6713.t1
PvAMP70	QAVLKRLLA	9	3	0.82	Oxyopinin-4a	88.89	Pv_tr_6712.t1
PvAMP71	TKPYMLKDN	9	2	1.38	Cupiennin-2a	66.67	Pv_tr_4146.t1
PvAMP72	AKIPGKVVR	9	4	1.09	Cupiennin-2a	77.78	Pv_tr_4145.t1
PvAMP73	NKDFKKTLK	9	4	1.63	OxTx1	77.78	Pv_sp_260.t1
PvAMP74	FLSKMKEIA†	9	2	0.96	Lycocitin 1	88.89	Pv_tr_8904.t1
PvAMP75	RHLVKQQLS	9	3	1.12	M-lycotoxin-Hc1a	87.5	Pv_tr_15055.t1
PvAMP77	LYSMIPKAV	9	2	0.97	AamAP1 [H8K]	66.67	Pv_so_8056.t1
PvAMP78	IPKALKNLI	9	3	0.82	AamAP1	77.78	Pv_tr_16804.t1
PvAMP79	PKKGLVRAI	9	4	1.09	AaeAP1 [S4.8.15K; A11K. N19K]	72.73	Pv_so_5175.t1
PvAMP80	VFKLFHKKK	9	5	1.79	Css54	77.78	Pv_sp_716.t1
PvAMP81	FSGNKLLPG	9	2	0.41	Im-5	80	Pv_so_3641.t1
PvAMP82	GRIFRLLRK	9	5	1.22	Scolopendin 2	88.89	Pv_so_3638.t1
PvAMP83	GRIGRLVLR	9	4	0.82	Scolopendin 2	88.99	Pv_sp_5222.t1
PvAMP84	ILKPFMLRR	9	4	0.95	Scolopin 2	77.78	Pv_sp_312.t1
PvAMP85	KPHHRTVTY	9	3	1.56	Gomesin (4-18) [Q9p]	77.78	Pv_so_1476.t1
PvAMP86	WSSIWAGMK	9	2	1.95	UyCT5	66.67	Pv_tr_75378t1
PvAMP87	IAALLKSIY	9	2	0.97	Meucin-13	66.67	Pv_sp_3763t1
PvAMP88	GPFWTGIKS	9	2	1.18	UyCT3 [L2F.S3G]. D1	66.67	Pv_sp_3762.t1
PvAMP89	LWQGSKSAI	9	2	1.32	Um3	77.78	Pv_tr_1841.t1
PvAMP91	GKLLEGLKK	9	3	1.36	UyCT1 [W7L.N12K]	88.89	Pv_sp_2709.t1
PvAMP92	LLDGVKKFI	9	2	0.82	UyCT1 [W7L.N12K]	85.71	Pv_tr_10673.t1
PvAMP95	DKCCDNCTRK	10	2	0.98	κ-theraphotoxin-Gr4c	62.5	Pv_sp_13803.t1
PvAMP164	RSVLKAHCRICRRRG	15	7	1.16	Androctonin	61.11	Pv_so_8460.t1
PvAMP169	CRRVCYKNRCVTYCRG†	16	6	1.47	Gomesin c(1-18)[Gln1]	87.5	Pv_sp_11662.t1
PvAMP170	CNKLRSDHFHLCNFQF	16	2	0.64	M-theraphotoxin-Gr1a	56.25	Pv_sp_11936.t1
PvAMP172	CRKLCFRNRCLTYCRGR	17	7	1.23	cGomesin	70.59	Pv_tr_7912.t1
PvAMP177	QCRKLCFRNRCLTYCRGR	18	7	1.24	Gomesin c(1-18)[Gln1]	72.22	Pv_tr_7912.t1
PvAMP179	VKMCRWTKSMLRGRGGCY	18	6	1.48	Androctonin	50	Pv_sp_11670.t1
PvAMP183	QCRKLCFRNRCLTYCRGRG	19	7	1.17	Gomesin c(1-18)[Gln1]	72.22	Pv_tr_7912.t1

Note: † Sequence predicted from the HMMER3 strategy. A: Peptide length. B. Peptide positive charge. C: Amphipathic index. D: Peptide identity percentage towards homologous peptide within the cDB.

Table S3. LC/MS analysis of *P. verdolaga's* AMPs.

AMP	Sequence	Theoric mass	m/z ratio						Observed ions	
			+1	+2	+3	+4	+5	+6		
PvAMP7	SLWGMWR-NH_2	934.12 Da	935.1	468.1	312.4	234.5	187.8	156.7	934.6	468.9
PvAMP32	IIKKIWK-NH_2	927.23 Da	928.2	464.6	310.1	232.8	186.4	155.5	464.6	310
PvAMP66	WKKIKKFF-NH_2	1123.43 Da	1124	562.7	375.5	281.9	225.7	188.2	375.4	
PvAMP69	YRARCVIYC-NH_2	1145.41 Da	1146	573.7	382.8	287.4	230.1	191.9	573.5	
PvAMP82	GRIFRLLRK-NH_2	1157.46 Da	1159	579.7	386.8	290.4	232.5	193.9	579.7	386.7
PvAMP164	RSVLKAHCRICRRRG-NH_2	1810.22 Da	1811	906.1	604.4	453.6	363	302.7	604	453.3
PvAMP172	CRKLCFRNRCLTYCRGR-NH_2	2147.63 Da	2149	1074.8	716.9	537.9	430.5	358.9	716.9	
PvAMP177	QCRKLCFRNRCLTYCRGR-NH_2	2275.76 Da	2277	1138.9	759.6	569.9	456.2	380.3	570.1	457
PvAMP183	QCRKLCFRNRCLTYCRGRG-NH_2	2332.82 Da	2334	1167.4	778.6	584.2	467.6	389.8	583.5	467

Table S4. IC_{50} values for isobologram analysis.

Gentamicin (μM)	50	25	12.50	6.25	3.12	1.56	0.39	0.1
PvAMP66 (μM)	17.01<br>(10.01 - 23.19)	29.096<br>(25.59 - 32.62)	31.75<br>(26.89 - 37.26)	29.27<br>(26.52 - 32.23)	9.85<br>(7.81 - 11.89)	15.53<br>(13.50 - 17.77)	NC	NC

Note: In parentheses 95% confidence intervals. NC = Logistic or linear regression with Pearson's correlation coefficient (R^2) < 0.70.

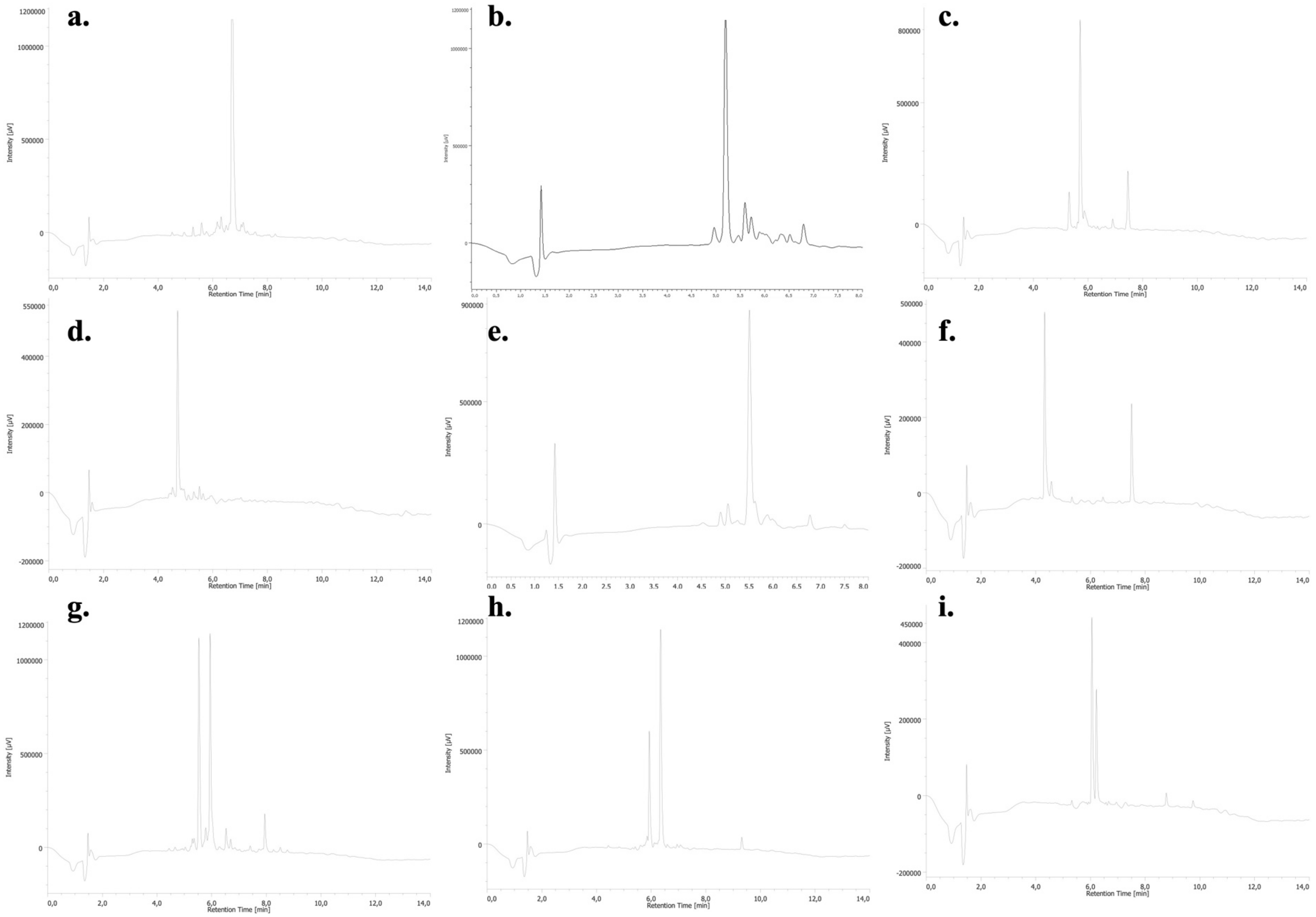

Figure S1. RP-HPLC analysis of *P. verdolaga's* AMPs: (**a**) Peptide PvAMP7. (**b**) Peptide PvAMP32. (**c**) Peptide PvAMP66. (**d**) Peptide PvAMP69. (**e**) Peptide PvAMP82. (**f**) Peptide PvAMP164. (**g**) Peptide PvAMP172. (**h**) Peptide PvAMP177. (**i**) Peptide PvAMP183.

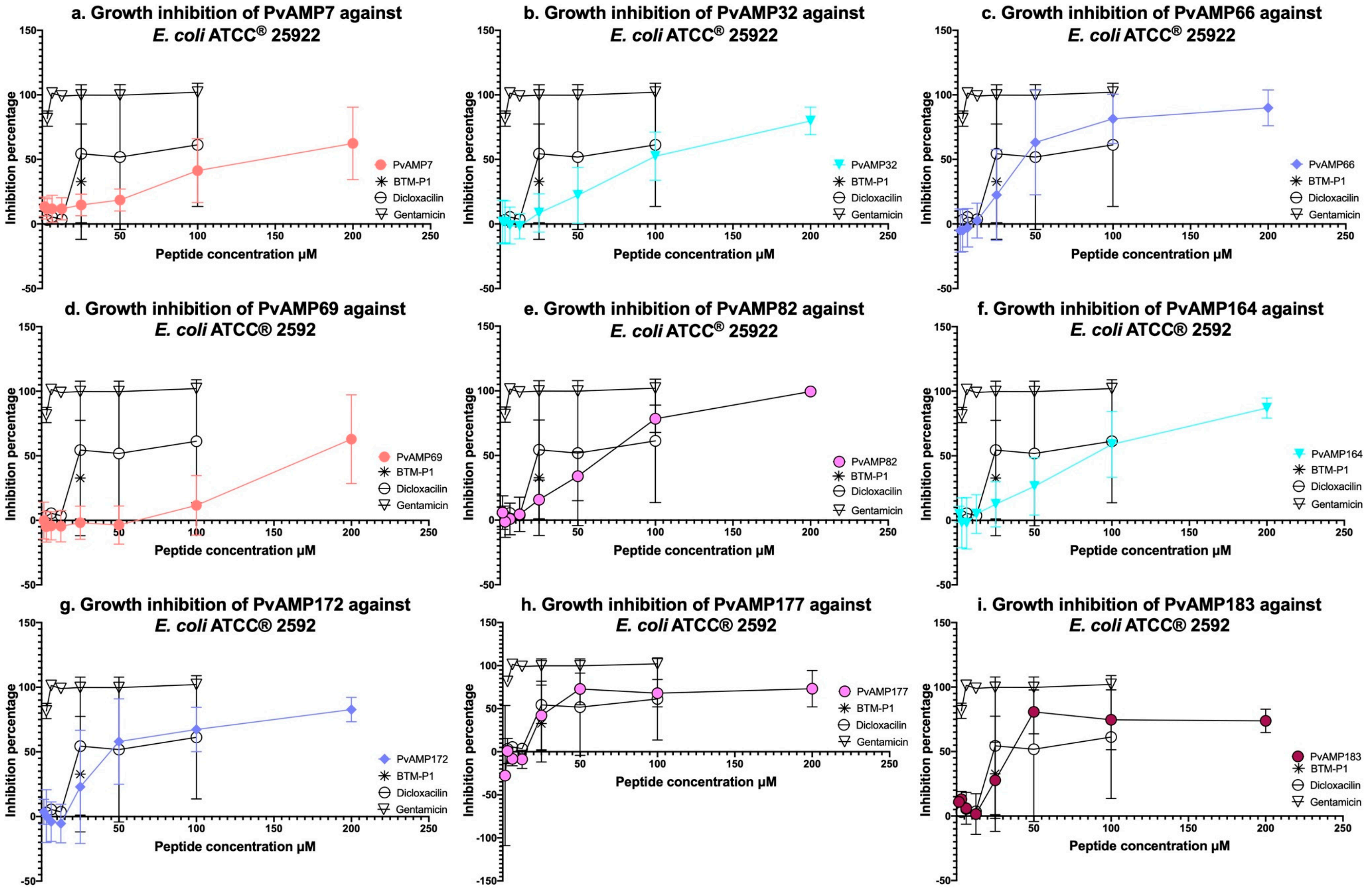

Figure S2. Dose-response curves of *P. verdolaga's* AMPs against *E. coli* ATCC® 25922: (**a**) PvAMP7; (**b**) PvAMP32; (**c**) PvAMP66; (**d**) PvAMP69; (**e**) PvAMP82; (**f**) PvAMP164; (**g**) PvAMP172; (**h**) PvAMP177; (**i**) PvAMP183.

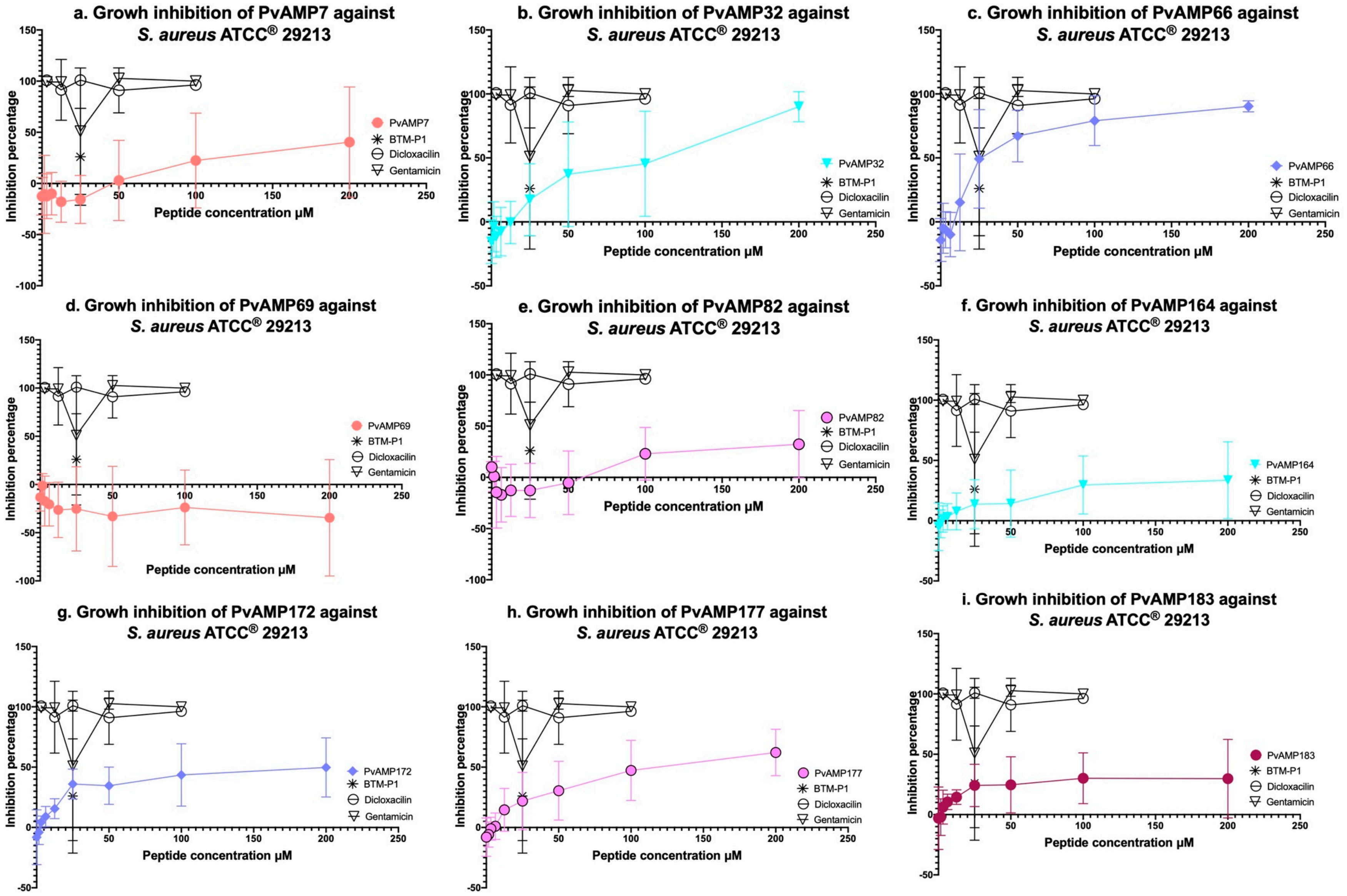

Figure S3. Dose-response curves of *P. verdolaga's* AMPs against *S. aureus* ATCC® 29213: (**a**) PvAMP7; (**b**) PvAMP32; (**c**) PvAMP66; (**d**) PvAMP69; (**e**) PvAMP82; (**f**) PvAMP164; (**g**) PvAMP172; (**h**) PvAMP177; (**i**) PvAMP183.

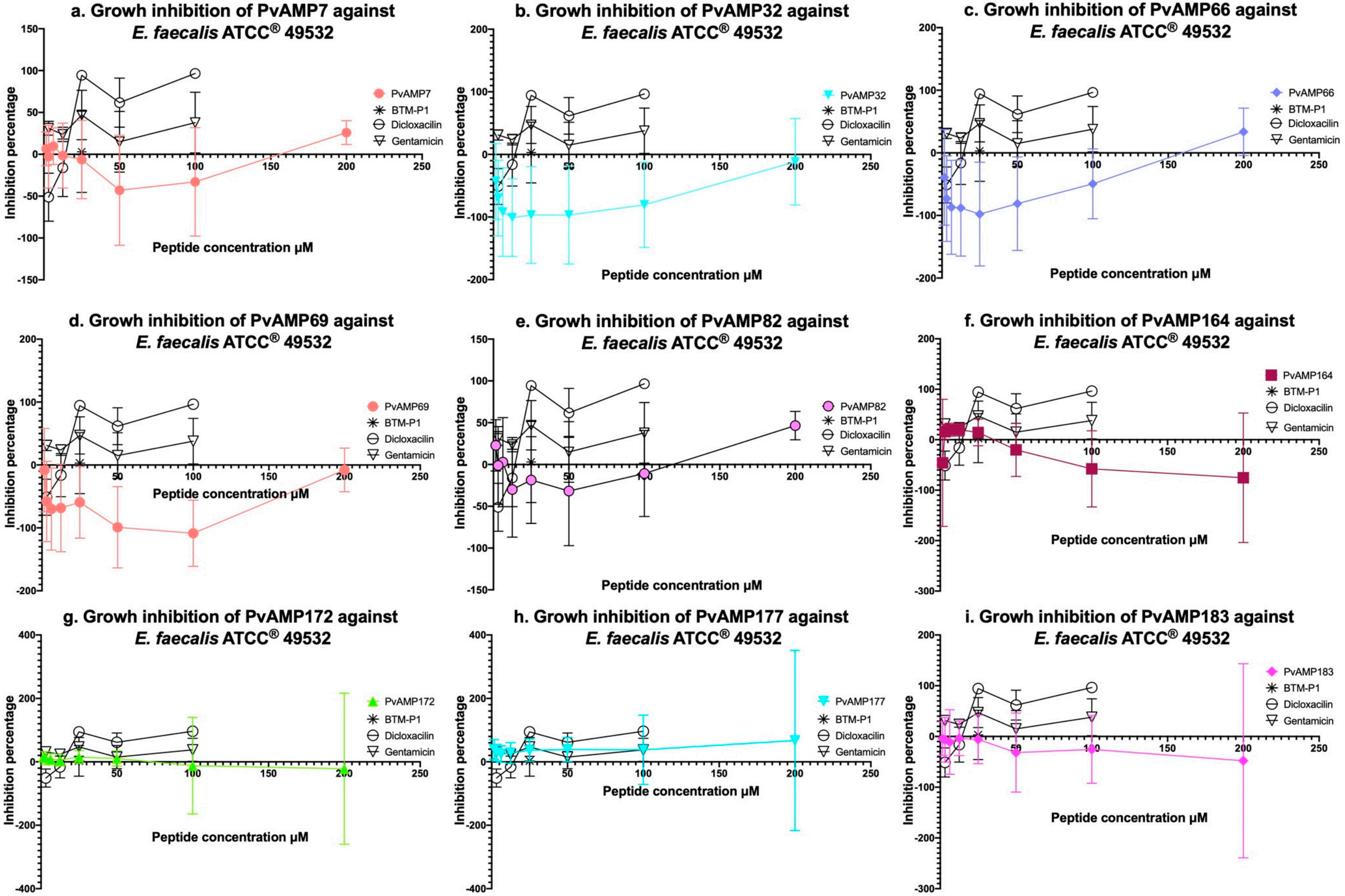

Figure S4. Dose-response curves of *P. verdolaga's* AMPs against *E. faecalis* ATCC® 49532: (**a**) PvAMP7; (**b**) PvAMP32; (**c**) PvAMP66; (**d**) PvAMP69; (**e**) PvAMP82; (**f**) PvAMP164; (**g**) PvAMP172; (**h**) PvAMP177; (**i**) PvAMP183.

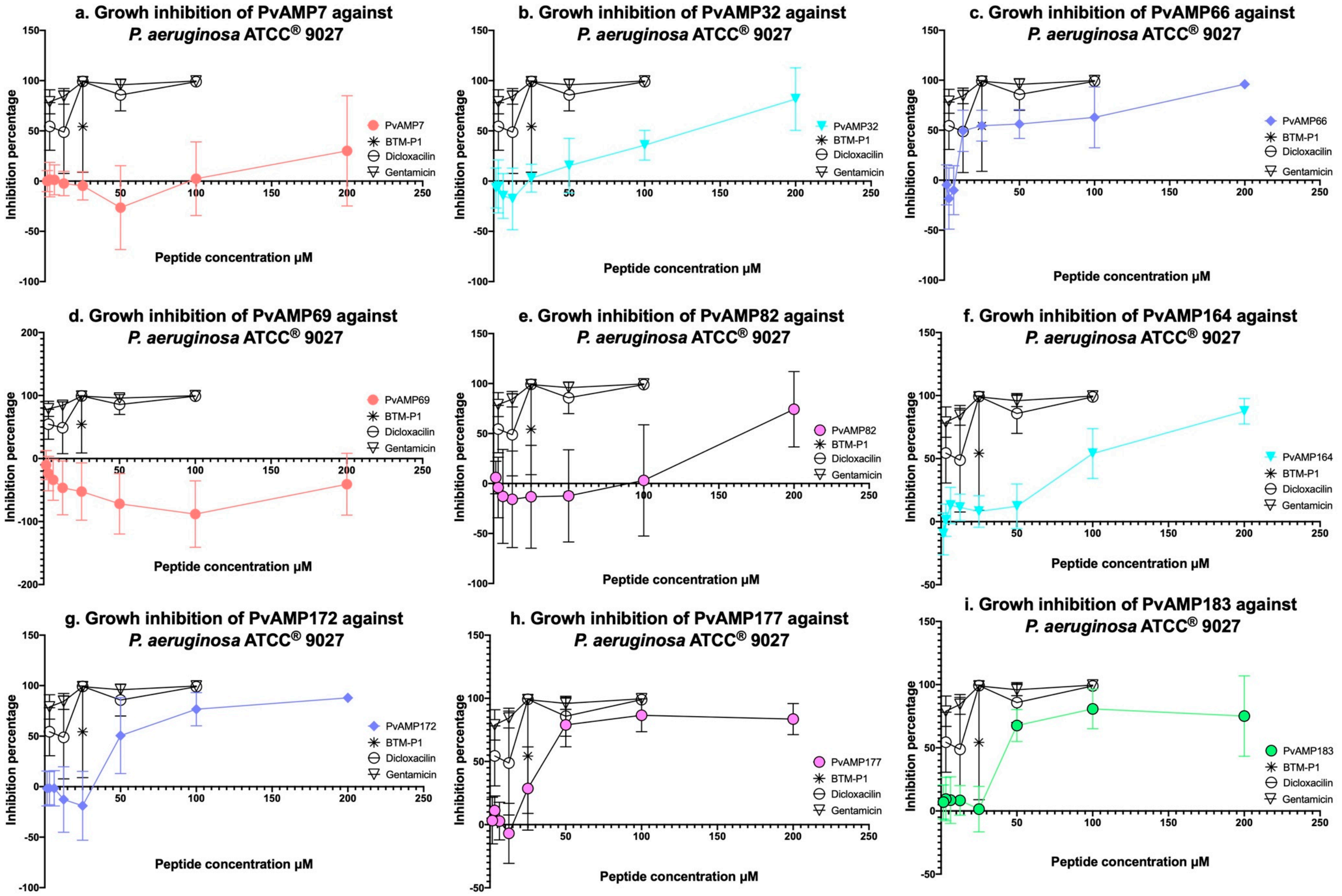

Figure S5. Dose-response curves of *P. verdolaga's* AMPs against *P. aeruginosa* ATCC® 9027(**a**) PvAMP7; (**b**) PvAMP32; (**c**) PvAMP66; (**d**) PvAMP69; (**e**) PvAMP82; (**f**) PvAMP164; (**g**) PvAMP172; (**h**) PvAMP177; (**i**) PvAMP183.

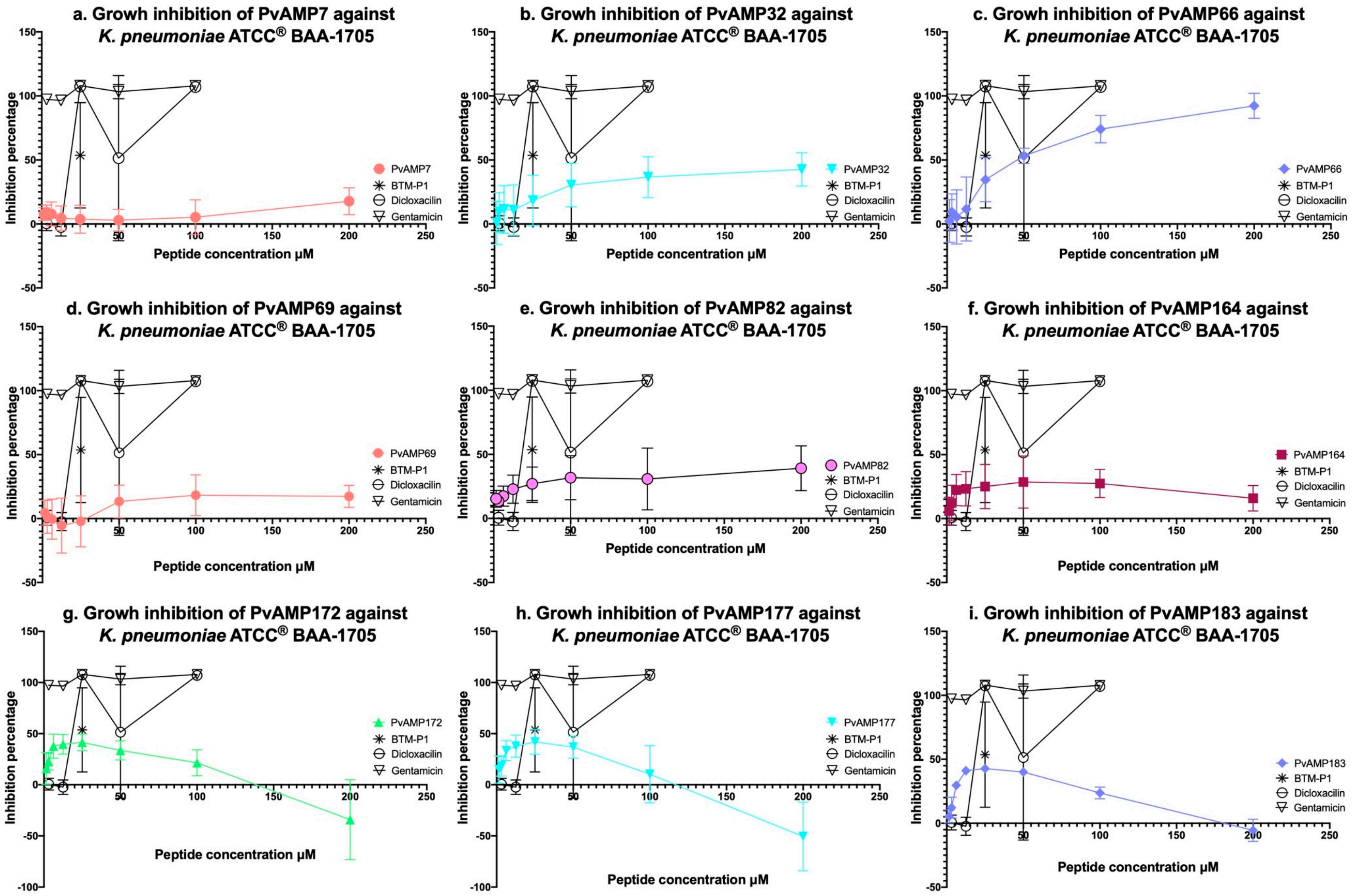

Figure S6. Dose-response curves of *P. verdolaga's* AMPs against *K. pneumoniae* ATCC® BAA-1705: (**a**) PvAMP7; (**b**) PvAMP32; (**c**) PvAMP66; (**d**) PvAMP69; (**e**) PvAMP82; (**f**) PvAMP164; (**g**) PvAMP172; (**h**) PvAMP177; (**i**) PvAMP183.

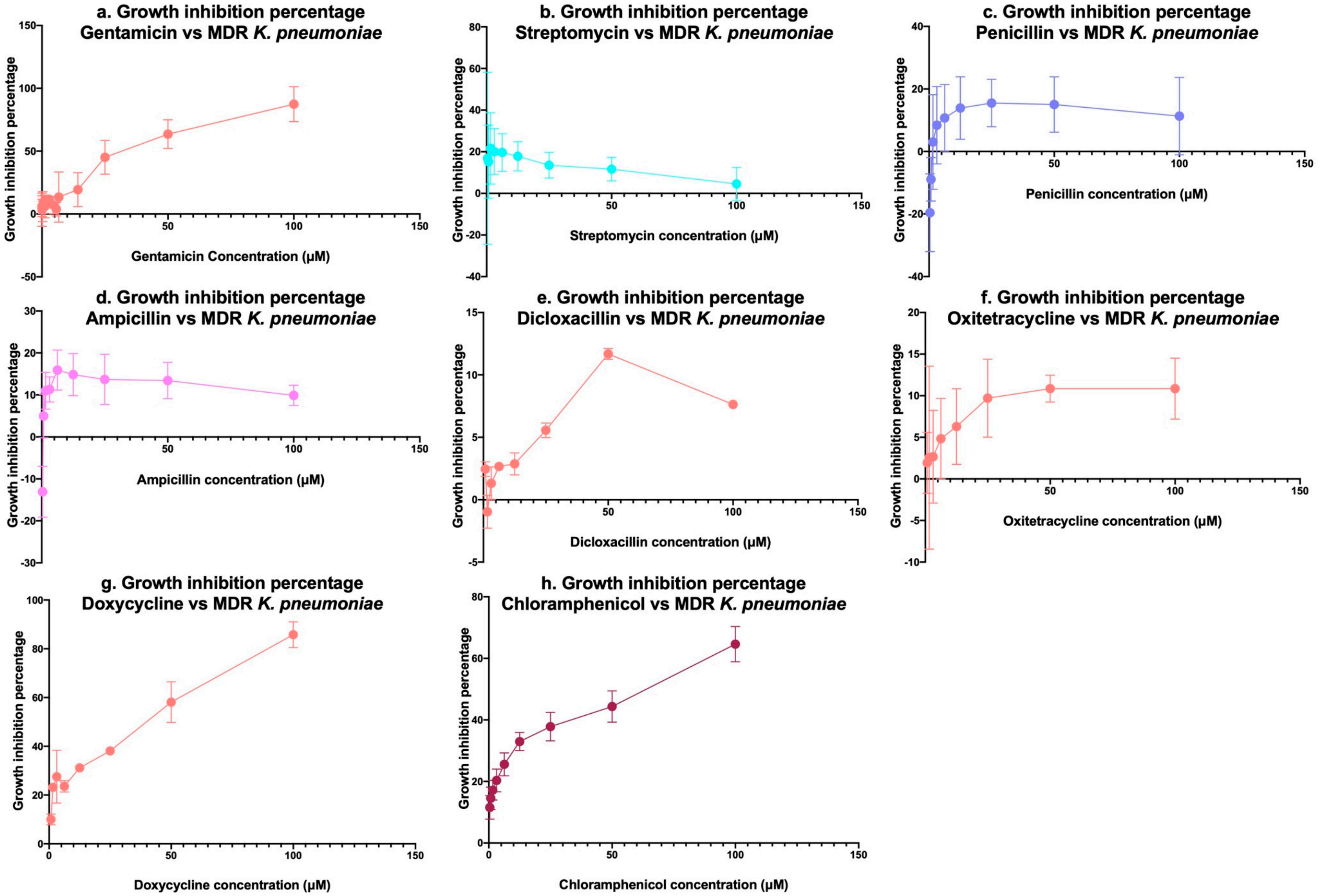

Figure S7. Dose-response curves of MDR *K. pneumoniae* against: (**a**) Gentamicin; (**b**) Streptomycin; (**c**) Penicillin; (**d**) Ampicillin; (**e**) Dicloxacillin; (**f**) Oxytetracycline; g(**g**) Doxycycline; (**h**) Chloramphenicol.

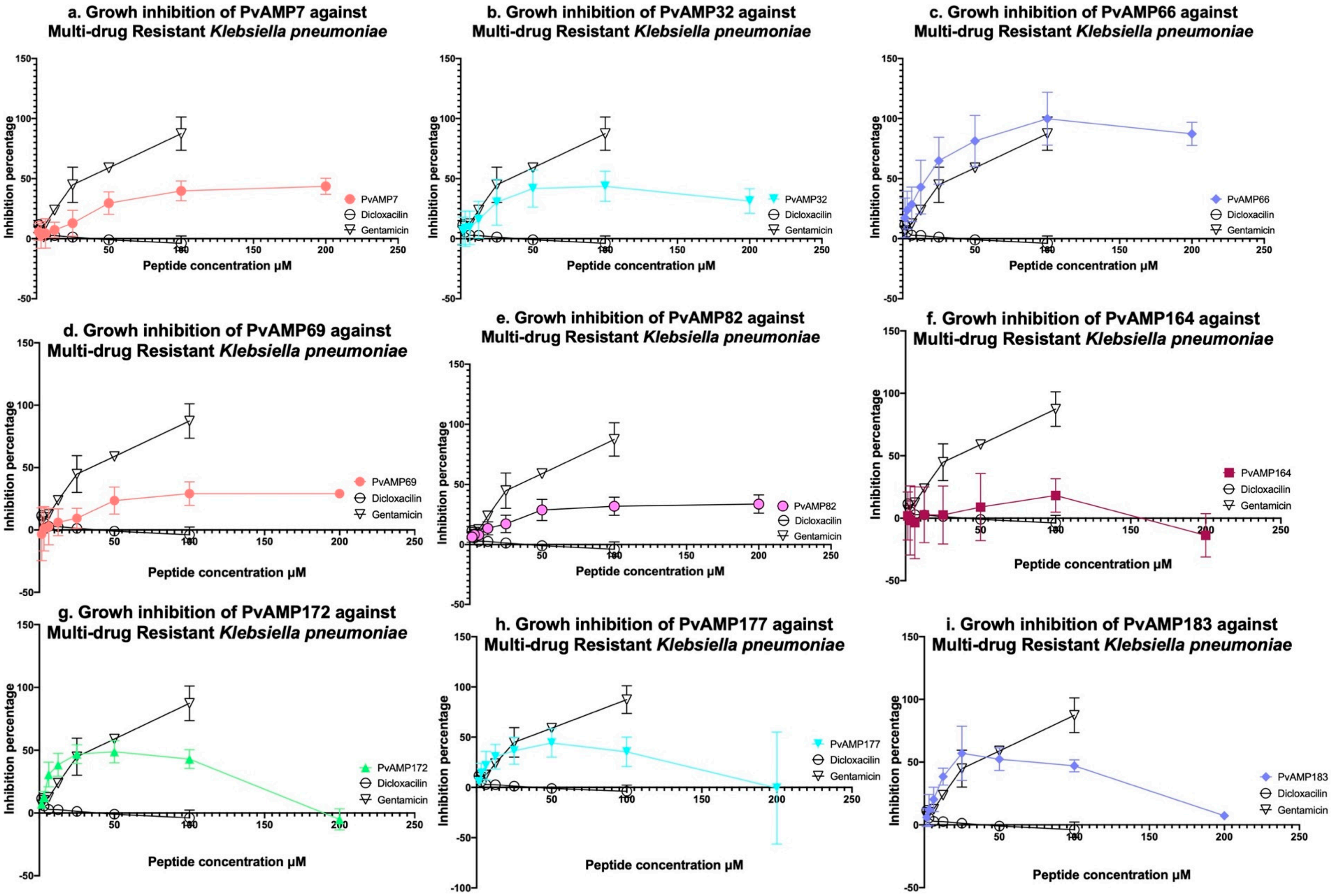

Figure S8. Dose-response curves of *P. verdolaga's* AMPs against MDR *K. pneumoniae* (**a**) PvAMP7; (**b**) PvAMP32; (**c**) PvAMP66; (**d**) PvAMP69; (**e**) PvAMP82; (**f**) PvAMP164; (**g**) PvAMP172; (**h**) PvAMP177; (**i**) PvAMP183.

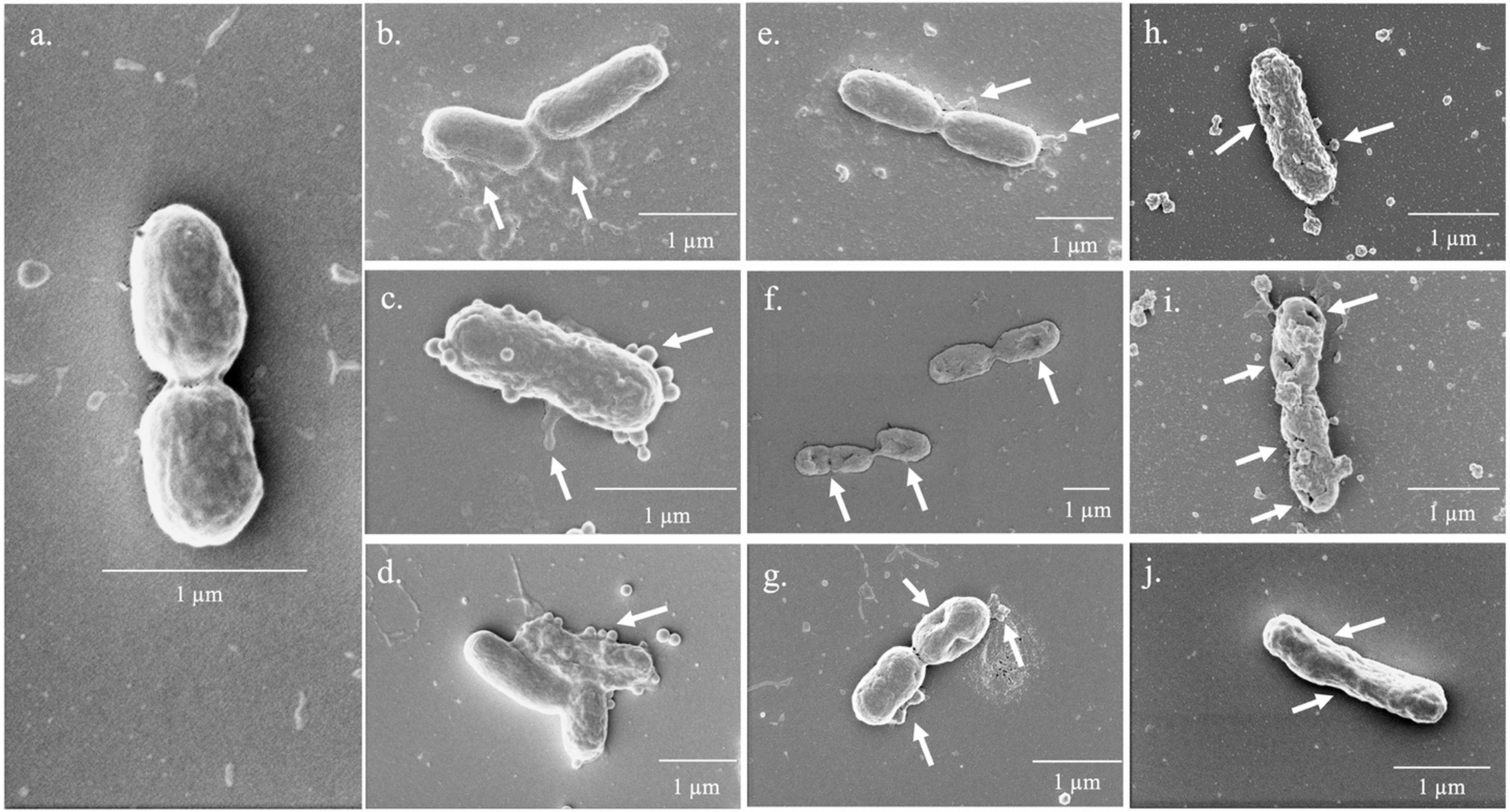

Figure S9. SEM analysis: (**a**) the multidrug resistant *K. pneumoniae* strain in media; and against (**b**) P7; (**c**) P32; (**d**) P66; (**e**) P69; (**f**) P82; (**g**) P164; (**h**) P172; (**i**) P177; (**j**) P183.

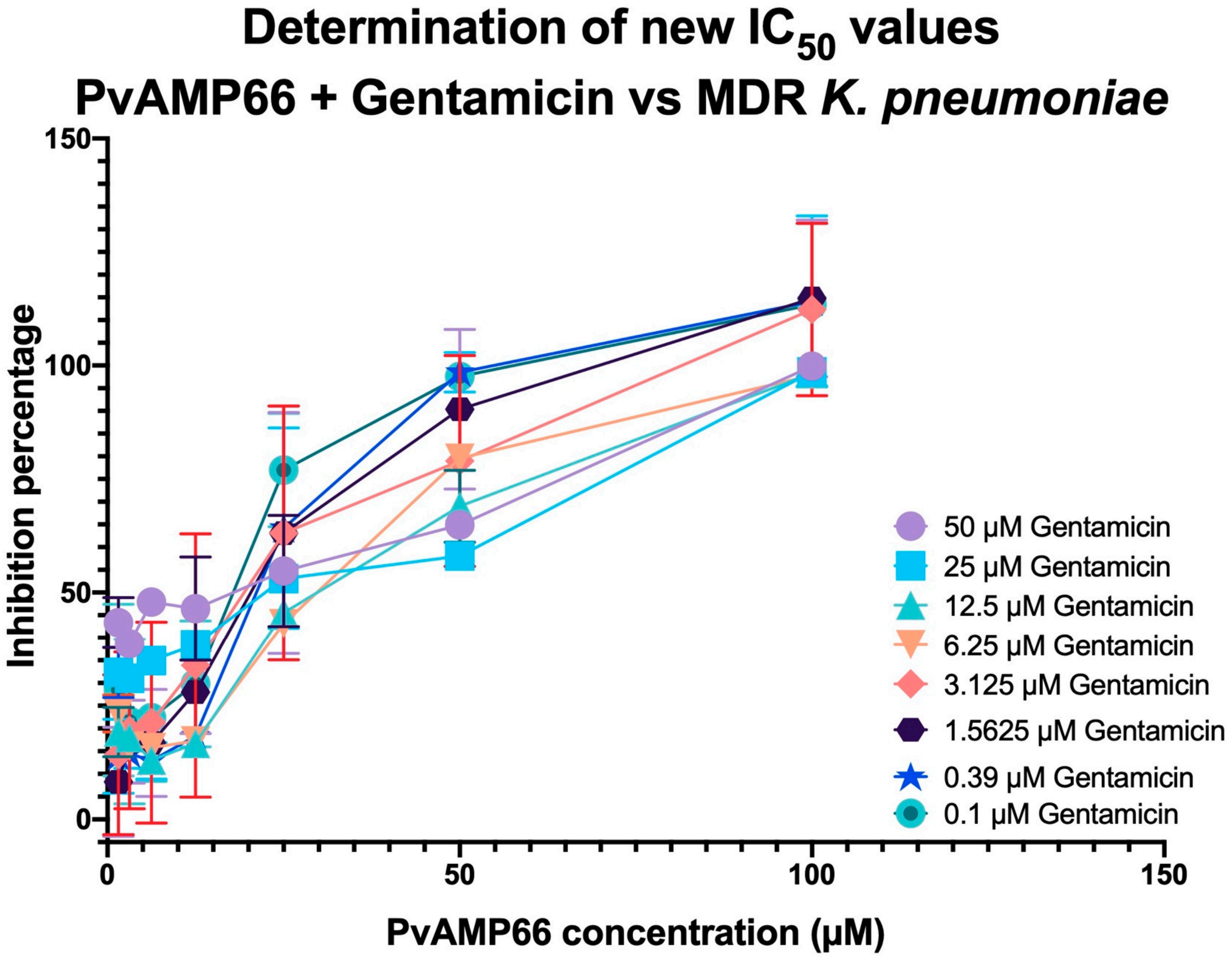

Figure S10. Dose-response curves of the isobologram analysis
